# Structure and function analyses of the Mmd2 gene in pacific white shrimp *Litopenaeus vannamei*

**DOI:** 10.3389/fgene.2023.1151193

**Published:** 2023-07-07

**Authors:** Shuqing Si, Xiaojun Zhang, Yang Yu, Xiaoxi Zhang, Xiaoyun Zhong, Jianbo Yuan, Song Yang, Fuhua Li

**Affiliations:** ^1^CAS and Shandong Province Key Laboratory of Experimental Marine Biology, Institute of Oceanology, Chinese Academy of Sciences, Qingdao, China; ^2^ School of Life and Sciences, Qingdao Agricultural University, Qingdao, China; ^3^ Key Laboratory of Breeding Biotechnology and Sustainable Aquaculture, Chinese Academy of Sciences, Wuhan, China; ^4^ Center for Ocean Mega-Science, Chinese Academy of Sciences, Qingdao, China; ^5^ University of Chinese Academy of Sciences, Beijing, China

**Keywords:** *Litopenaeus vannamei*, MMD2, PAQR10, gene structures, expression profile, growth

## Abstract

Monocyte to macrophage differentiation factor 2 gene (*Mmd2*) encodes a member of the progestin and adipoQ receptor (PAQR) family, and plays a key role in growth and development. Our previous studies had found *Mmd2* (Monocyte to macrophage differentiation factor 2) is a new candidate gene for growth traits in Pacific white shrimp (*Litopenaeus vannamei*). For the purpose of understanding the underlying mechanism of *LvMmd2* affecting the growth of shrimp, we analyzed the gene structure, phylogeny, expression profiles and RNA interference of this gene in *L. vannamei*. We found the LvMmd2 gene sequence was highly conserved in transmembrane regions, it was widely expressed in different tissues, with the highest expression level in the eye stalk. Knockdown *LvMmd2* could significantly promote body length and body weight gain, suggesting it is a growth suppressor. Through transcriptome analysis we identified 422 differentially expressed genes (DEGs) between the dsMmd2 group and control group, among which 337 genes were upregulated in the dsMmd2 group, including numerous muscle-related genes and protein synthesis genes. Further bioinformatics analysis showed that growth, metabolism, and immune-related signal pathway had changed significantly. The above results greatly increase our understanding on the conservative structure and function of LvMmd2 gene, and provide potential application prospects in genetics and breeding.

## 1 Introduction

The Pacific white shrimp (*Litopenaeus vannamei*) is one of the most economically important marine crustacean species in the world. At present, studies on the growth traits of shrimp mainly focus on screening growth-related markers and identification of quantitative trait loci (QTLs) or genes (Liu and Cordes, 2004; Yu et al., 2019). Through QTL mapping and related studies, a number of genes related to shrimp growth have been discovered, such as elongation of very long chain fatty acids-like (*ELOVL*) (Lyons et al., 2007), *AMY2* and *CTSL* (Glenn et al., 2005), *myostatin* (Zhuo et al., 2017; Kong et al., 2020), *myosin* (Kamimura et al., 2008), *PKC-delta* and *Rap-2a* (Yu et al., 2019), *LvSRC* (Wang Q. et al., 2019), *LvMMD2* (Wang et al., 2020), *dCMPD* and *NPTK* (Lyu et al., 2021), etc. However, the growth trait of shrimp is a polygenic trait controlled by multiple QTLs and numerous signal pathways, there is still a long way to elucidate the genetic mechanisms of shrimp growth.

In our previous studies, by Genome-Wide Association Studies (GWAS) analysis and QTLs mapping research, an SNP ref-259780-6 in the intron region of *LvMmd2*, showed a powerful association with the body weight of *L. vannamei* (Wang et al., 2020). So the monocyte to macrophage differentiation factor 2 (*Mmd2*) gene, encoding Progestin and AdipoQ Receptor 10 (PAQR10), was preliminarily identified as a growth-related gene. PAQR10 has an ancient seven transmembrane motif and is a member of the progestin and AdipoQ receptor (PAQR) family (Tang et al., 2005; Thomas et al., 2007). The PAQR family consists of 11 membrane receptors that are widely present in eukaryotes and some eubacteria, and exhibit a high conservation (Tang et al., 2005), indicating that this family may exert important biological functions.

PAQR family members play regulatory roles in fatty acid oxidation, inflammatory reaction, metabolic process, apoptosis, tumorigenesis, sex hormone regulation, etc (Svensk et al., 2013; Huang et al., 2021). There are many researches on these family members, including PAQR1, PAQR2, PAQR5, PAQR7, and PAQR8. PAQR1 and PAQR2 are key proteins that mediate the biological effects of adiponectin (Hosch et al., 2006). PAQR3 is structurally similar to PAQR1 and PAQR2, so it belongs to the adiponectin related receptor subgroup (Feng et al., 2007; Garitaonandia et al., 2009). PAQR5, PAQR7, and PAQR8 are also called progesterone membrane receptor mPRα, mPRβ, and mPRγ, they are involved in the rapid regulation of progesterone (Fernandes et al., 2005). PAQR6 and PAQR9 belong to the progesterone related membrane protein subgroup (Fernandes et al., 2005). At present, there is little research on PAQR10, the structure of PAQR10 and PAQR11 is similar to that of hemolysin III protein in bacteria. As a perforation membrane protein, the hemolysin protein uses mitochondria as the target organelle to mediate cell apoptosis, so it is speculated that PAQR10 may affect the process of cell apoptosis (Góñez et al., 2008). A subcellular localization experiment showed that PAQR10 was located on mitochondria, expressed in pancreas islet cells of adult rats, and the effect of PAQR10 on islets β-cell had played a role in promoting development and survival (Góñez et al., 2008). The homologue of PAQR10, PAQR11, plays an important role in early heart development of zebrafish embryos (Huang et al., 2012). Although we have learned that Mmd2 gene encodes the PAQR10 protein and may affect shrimp growth, the function and regulation mechanism of Mmd2 gene in shrimp are still unclear. Therefore, studying the function and mechanism of the Mmd2 gene of *L. vannamei* is a key to utilize this growth candidate gene.

In this research, we further confirmed the LvMmd2 gene and conducted a comprehensive analysis of its gene structure, expression profile, and function. Subsequently, we conducted RNA interference experiment, and found that dsRNA injection of *LvMmd2* could significantly improve the growth rate of shrimp. Comparative transcriptome analysis based on high-throughput sequencing was performed on the dsMmd2 group and control group, a number of differentially expressed genes (DEGs) were identified, we found that *LvMmd2* might play an important role in regulating muscle development and immunity. This study will elucidate the reliable connection between *LvMmd2* and shrimp growth, and will help us to understand the growth mechanism of shrimp, which may provide important basic information for utilizing *LvMmd2* as a growth marker for selective breeding.

## 2 Materials and methods

### 2.1 Experimental animals

The experimental shrimp, *L. vannamei*, were cultured in 25°C ± 1°C circulating seawater in our laboratory, and fed three times a day with equal weight commercial feed pellets. The shrimp with the body weight of 3.48 ± 1.82 g, and the body length of 6.45 ± 1.55 cm were used for RNA interference experiments. We guarantee that no conserved animals were used in this study.

### 2.2 Sequence analyses

To elucidate LvMmd2 gene structure and expression patterns, we scanned the gene in several transcriptomes previously reported (Wei et al., 2014; Gao et al., 2015; Wang F. et al., 2019; Zhang et al., 2019). Genome sequences data have been deposited in NCBI GenBank with the accession codes of QCYY00000000 (Zhang et al., 2019). The Early Development stages transcriptomic datasets were deposited in NCBI with accession numbers of SRR1460493-SRR1460505 (Wei et al., 2014). The early stage of WSSV infection transcriptomic datasets analyzed during the current study are available in the NCBI with the accession numbers SRR8149799-SRR8149804 (Wang F. et al., 2019). The Molting stages transcriptomic datasets from this article have been deposited in NCBI with accession numbers of SRX1098368-SRX1098375 (Gao et al., 2015). The obtained sequences were compared by the *L. vannamei* Genome Database (http://www.shrimpbase.net/vannamei.html) and BioEdit program (https://bioedit.software.informer.com/7.2/), redundant sequences were removed. The *LvMmd2* cDNA sequences were submitted to ORF Finder (https://www.ncbi.nlm.nih.gov/orffinder/) and locate their entire ORF region. The deduced amino acid sequences were obtained using ExPASy translation tool (http://web.expasy.org/translate/). Then, their amino acid sequences were analyzed by SMART (http://smart.embl-heidelberg.de/) to confirm the conserved domains.

### 2.3 Phylogenetic analyses

Firstly, we obtained multiple invertebrate and vertebrate Mmd2 proteins from the NCBI database to construct phylogenetic trees (Supplementary Table S1), including 31 protein sequences from arthropods (crustacean and insect), cnidarian, echinoderm, vertebrates, yeast and bacterium. Secondly, we constructed a phylogenetic tree of Mmd2 using MEGA X (http://www.megasoftware.net/) software. All Mmd2 sequences were aligned with priority program using the MUSCLE algorithm in MEGA X software and constructed the phylogenetic tree using the maximum likelihood (ML) distance algorithm of the priority program. Ultimately, the online website Tree of Life (iTOL) (https://itol.embl.de/) was used to visualize and decorate the constructed phylogenetic tree.

### 2.4 Expression analyses

In previous studies, we had accomplished RNA-Seq sequencing of twenty early development stages, eight molting stages, sixteen adult tissues and infection with three pathogens in *L. vannamei* (Wei et al., 2014; Gao et al., 2015; Wang F. et al., 2019; Zhang et al., 2019). We used the RSEM program (version1.3.0) (https://deweylab.github.io/RSEM) to further process transcriptome data, mapped clean reads to unigenes, and then calculated the fragments per kilo bases per million read (FPKM) values of *LvMmd2*. Finally, the calculated FPKM values were processed and compared the difference in expression of all unigenes. The data were normalized by log2 conversion and used to draw heatmaps with TBtools program (https://github.com/CJ-Chen/TBtools).

### 2.5 RNA isolation and cDNA synthesis

RNAiso Plus (TaKaRa, Japan) reagent was used to extract the total RNA from 15 tissues of *L. vannamei* following the manufacturer’s protocol. Then, the concentration and quality of extracted RNA were assessed by Nanodrop 2000 (Thermo Fisher Scientific, United States) and electrophoresis on 1% agarose gel. For synthesizing cDNA, 1 µg total RNA was used as the template, the PrimeScript RT Reagent Kit (TaKaRa, Japan) was used for reverse transcription synthesis of cDNA samples. The first step was to remove the genomic DNA (gDNA) using gDNA eraser, and the second step was to synthesize cDNA samples using PrimeScript RT Enzyme. Finally, the qualified cDNA samples were saved at −80°C.

### 2.6 Real-time quantitative PCR assay

18S rRNA was used as the internal reference gene and used SYBR Green-based qRT-PCR to detect the expression levels of *LvMmd2* and other genes. All primers were designed by Primer3Plus (http://www.primer3plus), as shown in Supplementary Table S3. An Eppendorf Mastercycler ep realplex (Eppendorf, Hamburg, Germany) was used for qRT-PCR. The single sample contained 5 μL SuperReal PreMix Plus, 0.6 mM primers (upstream primer and downstream primer), 3 μL DEPC water, 1 μL cDNA template, and Setted four technical repetitions for a single sample. The qPCR steps were as follows: 94°C for 2 min, 40 cycles of 94°C for 20 s, annealing temperature for 20 s, and 72°C for 20 s, the primer annealing temperature for each gene is shown in Supplementary Table S3. The specificity of the primers was detected by melting curve detection. Eventually, the formula of 2^−ΔΔCt^ method was used to calculate the relative expression level of objective genes (Livak and Schmittgen, 2001).

### 2.7 RNA interference of *LvMmd2*

Two pairs of primers, EGFP-F/R and *LvMmd2*-F/R were designed using Primer3Pluse, and the T7 promoter sequence was added to EGFP-F/R and *LvMmd2*-F/R form dsEGFP-F/R and dsMmd2-F/R (Supplementary Table S2). We use primers with T7 promoter sequence to amplify the target double-stranded RNA (dsRNA), the PCR was performed according to the manufacturers’ instructions of the Premix Ex Taq™ Hot Start Version (TaKaRa, Kyoto, Japan), the procedure was as follows: 95°C for 4 min, 40 cycles of amplification (94°C for 30 s, annealing temperature for 30 s, 72°C for extension time), 72°C for 10 min, annealing temperature and extension time are shown in Supplementary Table S2. The amplified PCR products were detected by electrophoresis on 1% agarose gel, the qualified PCR products were purified using the MiniBEST DNA fragment purification kit (TaKaRa, Maebashi, Gunma, Japan). We used the purified products as the template to synthesize dsRNA using a Transcript Aid T7 High Yield kit (Thermo Fisher Scientific, United States), then purify the synthesized dsRNA using a mixture of phenol and chloroform. The NanoDrop 2000 was used to check the concentration of dsRNA, redundant single-stranded RNA was removed by RNaseA (TaKaRa, Japan) and the quality of dsRNA was detected using 1.5% agarose electrophoresis. The qualified dsRNAs was stored at −80°C for subsequent RNA interference experiments.

To optimize the interference efficiency of *LvMmd2*, dsRNA was diluted with 10 µL PBS without RNA enzyme, three different injection dosages groups were set, 48 shrimps (3.40 ± 1.26 g each, in the premolt D1-D2 stage) were selected for the experiment and randomly divided into three groups (dsMMD2, dsEGFP, PBS). We injected three different doses of dsRNA (2, 6, and 10 µg) into the into the last abdominal segment of each shrimp, and injected the same doses of dsEGFP and PBS as the control group. After 48 h of dsRNA injection, the hepatopancreas of shrimp were sampled, three biological replicates were set in each group, and four technical replicates were set for each biological replicates, qRT-PCR was performed to detect interference efficiency. The result showed that 2 μg was the optimal interference dose, which could significantly reduce the expression level of *LvMmd2*.

We chose 2 μg for the following formal RNAi experiment, 480 shrimp individuals (3.48 ± 1.82 g each, in the premolt D1-D2 stage) randomly divided into three groups (dsMMD2, dsEGFP, PBS), and injected 2 μg of dsMmd2 and dsEGFP, 10 µL PBS, respectively. The same injection was repeated every 4 days, and the experiment lasted for 15 days. Before and after the experiment, the body weight and body length of every shrimp were measured and recorded. Then hepatopancreas and muscle tissues were sampled, preserved in liquid nitrogen, and long-term storage in −80°C.

### 2.8 Transcriptomic analysis of *LvMmd2* knockdown

To estimate the effect of *LvMmd2* knockdown in *L. vannamei*, a RNA-Seq analysis was performed to compare the dsMmd2 group with the control group. After the injection of 48 h, total RNA was extracted from the hepatopancreas of four individuals, three biological replicates were designed in each group (dsMmd2 group and EGFP control group). All total RNA were detected and evaluated as described above. RNA integrity was evaluated using the Agilent 2100 Bioanalyzer (Agilent Technologies, Santa Clara, CA, United States). Then, TruSeq Stranded mRNA LT Sample Prep Kit (Illumina, San Diego, CA, United States) was used to construct a sequencing library. The transcriptome sequencing was accomplished by OE Biotech Co., Ltd. (Shanghai, China).

The clean reads were mapped to the *L. vannamei* genome by HISAT2 (Kim et al., 2015). The FPKM was counted by Cufflinks (Trapnell et al., 2010), and the read counts were acquired by HTSeq-count (Anders et al., 2014). Differential gene expression analysis was undertaked by DESeq R package (Anders and Huber, 2013). We chosed *p*-value <0.05 and foldchange >2 as the threshold for differentially expressed genes (DEGs). The DEGs obtained were analyzed by hierarchical clustering analysis to prove the gene expression patterns in different samples. Gene ontology (GO) and Kyoto Encyclopedia of Genes and Genomes (KEGG) enrichment analyses of DEGs were undertaken respectively by R based on the hypergeometric distribution (Kanehisa et al., 2008).

### 2.9 Statistical analyses

We subjected to one-way ANOVA tests using SPSS in three or more groups of variables, and we used *t*-test for two groups of variables (https://www.ibm.com/cn-zh/analytics/spss-statistics-software) (version 20).

## 3 Results

### 3.1 Characteristics of *LvMmd2* sequence

**FIGURE 1:**
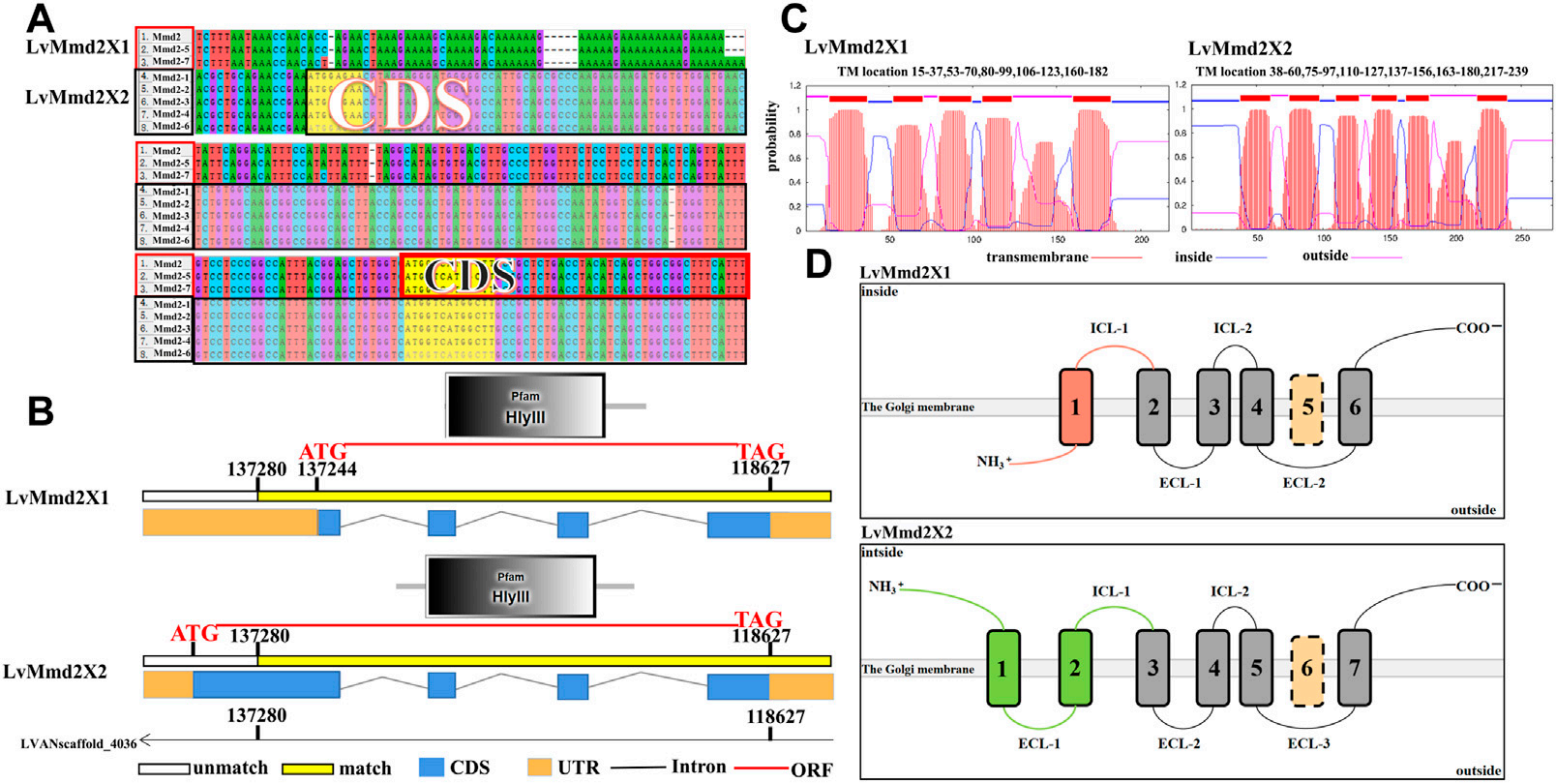
Gene structure of *LvMmd2*. **(A)** Sequence differences of the alternative splicing forms of *LvMmd2*. **(B)** Structure of the alternative splicing forms of *LvMmd2*. **(C)** Prediction of transmembrane structure of LvMmd2. **(D)** Schematic diagram of TM domain of LvMmd2.

A total of seven *LvMmd2* transcripts were identified from the *L. vannamei* RNA-Seq database, they are located in the same location of the genome LVANscaffold_4036. According to their gene structure maps, LvMmd2 gene contains two alternative splicing forms, which have been verified by PCR amplification and sequencing, named *LvMmd2X1* (XM_027379195.1) and *LvMmd2X2* (c83205_g1) (Supplementary Material) (Figure 1A). They differ in the first exon and its preceding 3′UTR sequence. The open reading frame (ORF) length of *LvMmd2X1* and *LvMmd2X2* is 654bp and 725bp, which encode 217aa, and 274aa respectively. The deduced LvMmd2X1 and LvMmd2X2 proteins have molecular weight (MW) of 24 kDa and 30 kDa, and isoelectric point (p*I*) of 6.22 and 6.35 (Table 1). Moreover, LvMmd2X1 protein has five highly conserved transmembrane (TM) domains, LvMmd2X2 protein has six highly conserved TM domains, and all of them have a conserved domain, Pfam-HLYIII domain (Figure 1A).

**TABLE 1:** Basic characteristics of *LvMmd2*.

Name	Gene length (bp)	Deduced aminoacids (aa)	Transmembrane (TM)	Molecular weight (kDa)	Isoelectric point (pI)	Signal peptide	Accession number	Location on genome
*LvMmd2X1*	3239	217	5	24.33	6.22	—	XM_027379195.1	LVANscaffold_4036
*LvMmd2X2*	3262	274	6	30.38	6.54	—	c83205_g1	LVANscaffold_4036

According to the transmembrane structural domain diagrams of the two alternative splicing forms, LvMmd2 protein is composed of a long and variable N-terminal region, the high conserved TMs, and a relatively conserved short C-terminal region (Figure 1B). Topology prediction analysis showed that LvMmd2X1 N-terminal region and extracellular loops (outside) confront the extracellular, whereas the C-terminal region and intracellular loops (inside) confront the cytoplasm. While LvMmd2X2 N-terminal region, C-terminal region and intracellular loops (inside) confront the cytoplasm, whereas the extracellular loops (outside) confront the extracellular (Figure 1B), this is unique to PAQR family members.

**FIGURE 2:**
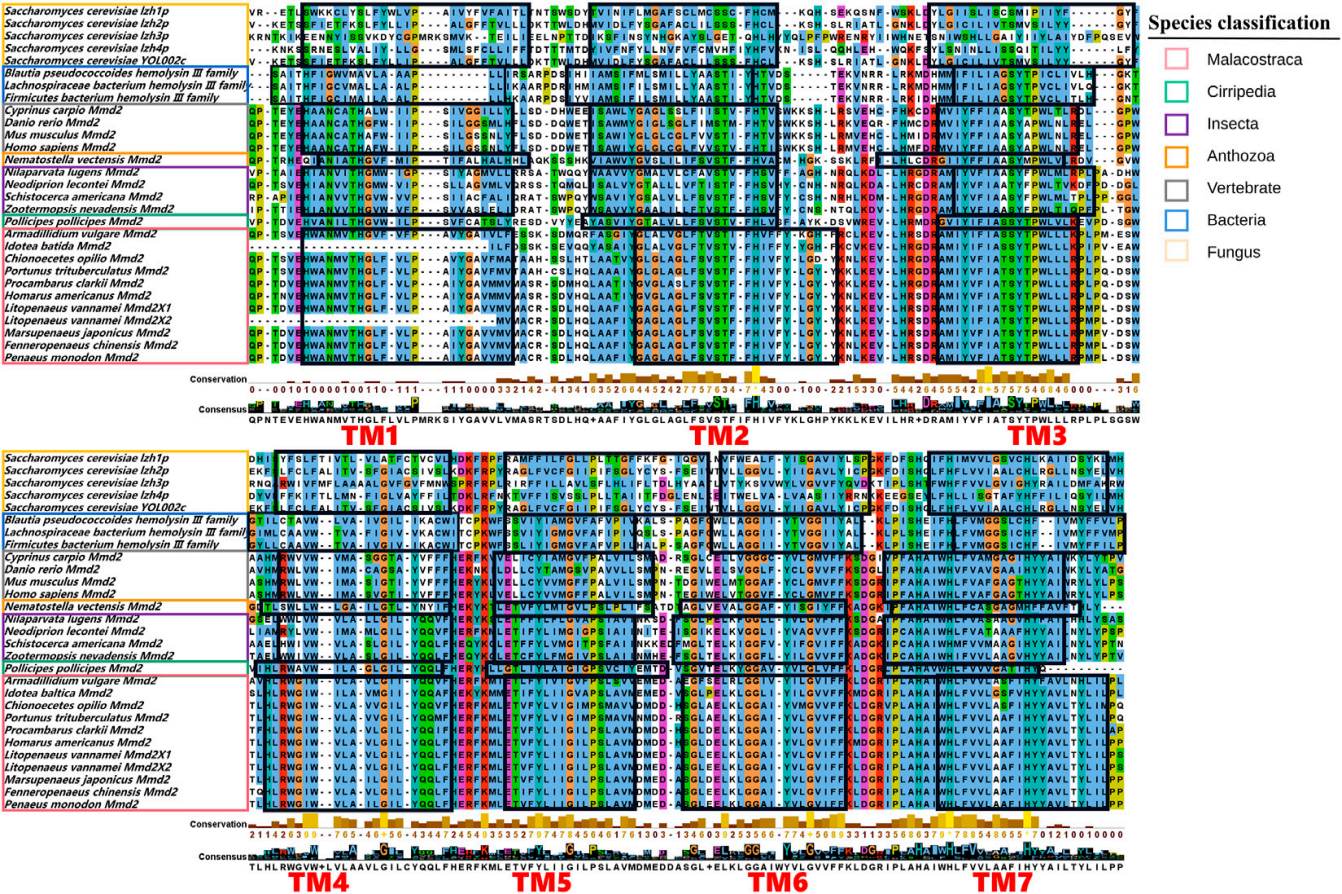
Multiple sequences alignment of the domains of the Mmd2 proteins. The conserved transmembrane domains are circled with black boxes and marked in red.

Multiple amino acid sequence comparison was performed to analyze the structural similarity among *LvMmd2* and Mmd2 genes from other different species (Supplementary Table S1). As showed in Figure 2, the transmembrane domain location and amino acid sequence length are different in these species. Except for bacteria and fungi, other most species lost TM1 or TM6, most vertebrates lost TM1, TM5, and TM6, while most invertebrates only lost TM1 and TM6, which is a specific difference between vertebrates and invertebrates (Table 2). Different species have distinct non-conservative N-terminal and C-terminal, and the overall amino acid sequence of bacteria and fungi is longer than other species.

**TABLE 2:** The loss of transmembrane (TM) domains of Mmd2 gene in different species.

Species		Gene	Missing domain
Vertebrate	*Cyprinus carpio*	*Mmd2*	TM6
	*Danio rerio*	*Mmd2*	TM5
	*Mus musculus*	*Mmd2*	TM1
Insecta	*Neodiprion lecontei*	*Mmd2*	TM6
	*Schistocerca americana*	*Mmd2*	TM6
Cirripedia	*Pollicipes pollicipes*	*Mmd2*	TM1 and TM6
Malacostraca	*Armadillidium vulgare*	*Mmd2*	TM6
	*Procambarus clarkii*	*Mmd2*	TM6
	*Homarus americanus*	*Mmd2*	TM6
	*Litopenaeus vannamei*	*LvMmd2X2*	TM6
	*Litopenaeus vannamei*	*LvMmd2X1*	TM1 and TM6

### 3.2 Phylogenetic analysis of Mmd2s

**FIGURE 3:**
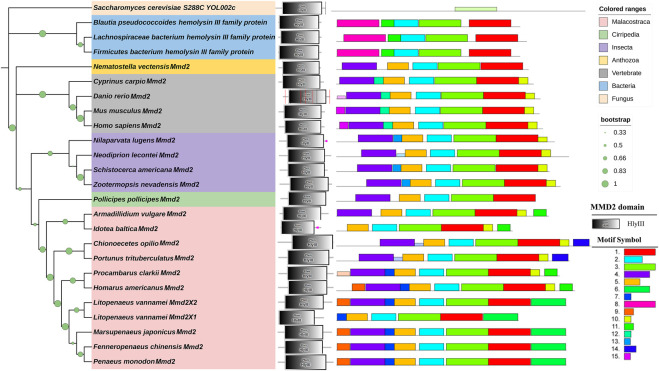
Phylogenetic tree of Mmd2s and their gene structures. The domains of Mmd2s obtained from the SMART program are located on the right of the phylogenetic tree. The bootstrap values are given at each branch node.

The LvMmd2 and Mmd2 sequences of other species obtained from the NCBI database (Supplementary Table S1) were used to construct the Mmd2 phylogenetic tree (Figure 3), which showed that LvMmd2 clustered with nine Malacostraca Mmd2 sequences, *Penaeus monodon*, *Fenneropenaeus chinensis*, *Marsupenaeus japonicus*, *Homarus americanus*, *Procambarus clarkii*, *Portunus trituberculatus*, *Chionoecetes opilio*, *Armadillidium vulgare*, *Idotea baltica*, this clade clusters together with the Cirripedia and Insecta. LvMmd2 has the same domain as other species, indicating that LvMmd2 is relatively conserved in the evolutionary process. By analyzing the domain of the Mmd2 protein in different species, it was found that bacteria, fungi and animals all had a typical HlyIII domain, indicating a high degree of homology of the Mmd2 gene exists in prokaryotes and eukaryotes. However, by comparing the motif of yeast and other species, it is found that the motif of yeast is different from that of other species. Although yeast has seven-transmembrane domains (Figure 2), its TM domains are not so conservative as those of other species.

### 3.3 Expression profiles of LvMmd2 gene

**FIGURE 4:**
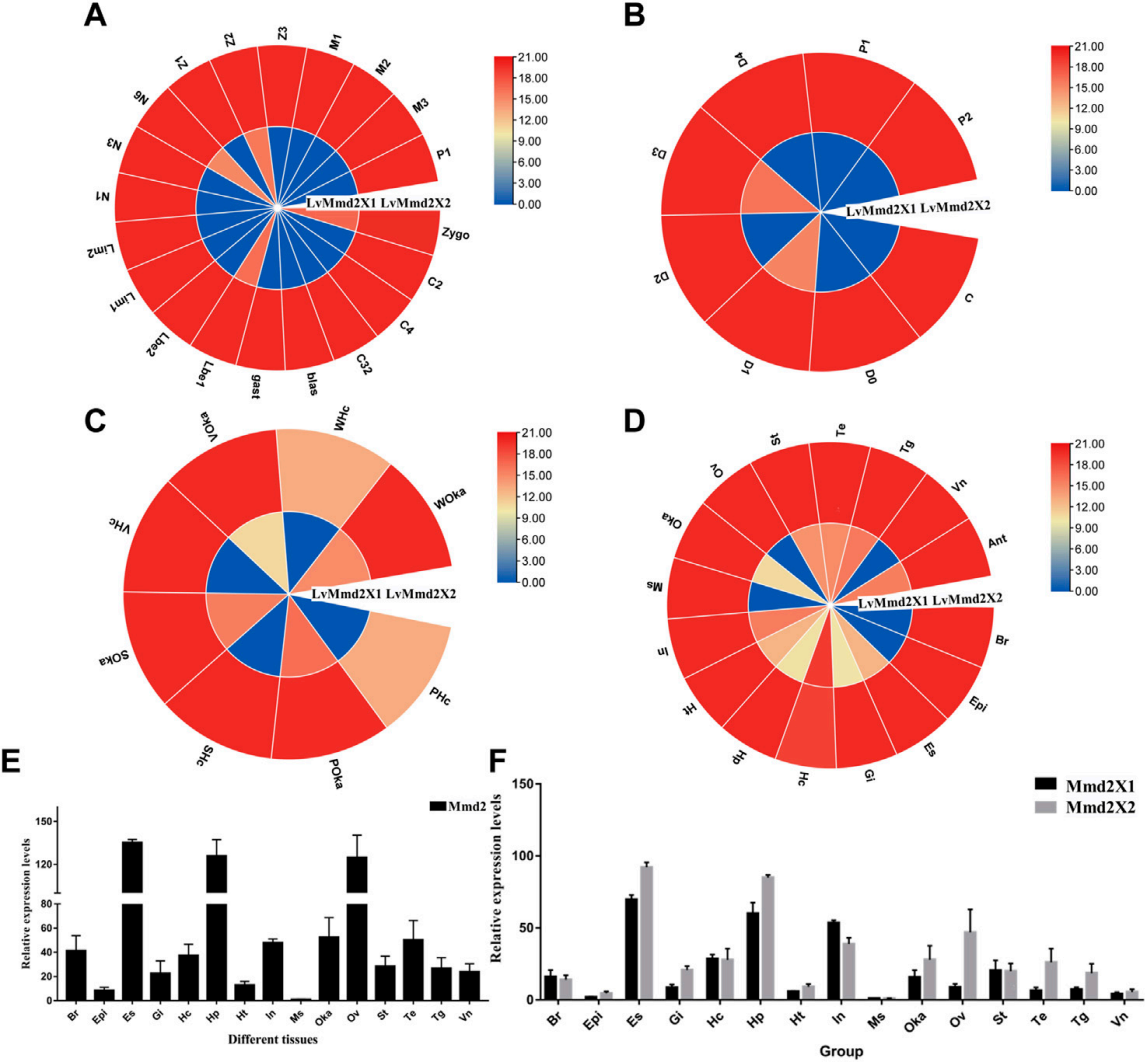
The expression profiles of LvMmd2 gene. **(A)** Early development stages: zygo (zygote), C2 (2 cells), C4 (4 cells), C32 (32 cells), blast (blastula), gast (gastrula), Lbe1 (limb bud embryo I), Lbe2 (limb bud embryo II), Lim1 (larva in membrane I), Lim2 (larva in membrane II), N1 (nauplius I), N3 (nauplius III), N6 (nauplius VI), zoea I Z1 (),Z2(zoea II), Z3 (zoea III), M1 (mysis I), M2 (mysis II), M3 (mysis III), and P1 (postlarvae 1) (Wei et al., 2014); **(B)** Molting stages: intermolting **(C)**, premolting (D0, D1, D2, D3, and D4), and postmolting (P1 and P2) stages (Gao et al., 2015); **(C)** Adult different tissues: Hc (hemocyte), Ant (antennal gland), Ms (muscle), In (intestines), Ov (ovary), St (stomach), Oka (lymphoid organ), Gi (gill), Hp (hepatopancreas), Te (testis), Es (eye stalk), Br (brain), Tg (thoracic ganglion), Vn (ventral nerve), Epi (epidermis), Ht (heart) (Zhang et al., 2019); **(D)** Bacterial infections: PBS (Phosphate Buffer Saline), S (*Staphylococcus aureus*), V (*Vibrio parahaemolyticus*), W (White spot syndrome virus) (Wang F. et al., 2019). These experiments and data are from previous RNA-Seq studies, respectively. **(E)** The expression of *LvMmd2* gene in different tissues was detected by qPCR. **(F)** The expression of *LvMmd2X1* and *LvMmd2X2* in different tissues was detected by qPCR.

By analyzing the RNA-Seq data of early development of *L. vannamei*, we found that *LvMmd2X1* was higher expressed in zygote (zygo), lamb bud embryo I (Lbe1), nauplius VI (N6), zoea II (Z2) stage, while *LvMmd2X2* was highly expressed throughout the early development stages (Figure 4A).

In the molting process, *LvMmd2X1* was low expressed before the premolt D0 stage and reached its maximum value at the D1 stage, and then the lowest value appeared at the D2 stage, and reached another small peak at the D3 stage. The expression level of *LvMmd2X2* was relatively high and the change range was not very dramatic (Figure 4B).

According to the transcriptional profile of *LvMmd2* after different pathogeny infections, *LvMmd2* probably plays an important role when infected with *Staphylococcus aureus* (Gram-positive bacterium), *Vibrio parahaemolyticus* (Gram-negative bacterium) and WSSV (virus). In hemocyte, *LvMmd2X2* expression was significantly up-regulated when infected by different pathogens, but in lymphoid (Oka) organ, the upregulated changes were not obvious compared with PBS control group. Additionally, the expression of *LvMmd2X1* was very low and no obvious changes when infected by different pathogens (Figure 4C).

By analyzing the RNA-Seq data of shrimp adult tissues, the expression of *LvMmd2* was determined. *LvMmd2* was expressed in every tissues, the expression level is highest in the eye stalk, followed by hepatopancreas, intestines, nervous system tissues, and lymphoid organ, the expression of *LvMmd2X2* was generally higher than that of *LvMmd2X1*, however, *LvMmd2X1* was mainly highly expressed in hemocyte, intestines, antennal gland, and thoracic ganglion (Figure 4D). Then, the expression patterns of *LvMmd2* in different tissues of *L. vannamei* were analyzed using qRT-PCR (primer was designed at the homologous region of *LvMmd2X1* and *LvMmd2X2* to detect all *LvMmd2* expression levels) among different tissues of *L. vannamei*, the expression levels of *LvMmd2* were detected in 15 tissues of shrimp. Comparison of RNA-Seq and qRT-PCR expression profiles, the eye stalk has the highest expression level among them, followed by ovary and hepatopancreas (Figure 4E). Furthermore, in order to explore whether the expression levels of *LvMmd2X1* and *LvMmd2X2* are different, primers were designed at differential splicing locations, and the expression of *LvMmd2X1* and *LvMmd2X2* genes in the 15 tissues of adult shrimp were detected by qRT-PCR again. They have the same expression pattern as whole *LvMmd2*, the expression of *LvMmd2X2* was significantly higher than that of *LvMmd2X1* in all detected tissues, except intestine, hemocyte and brain (Figure 4F).

### 3.4 RNA interference of *LvMmd2*

**FIGURE 5:**
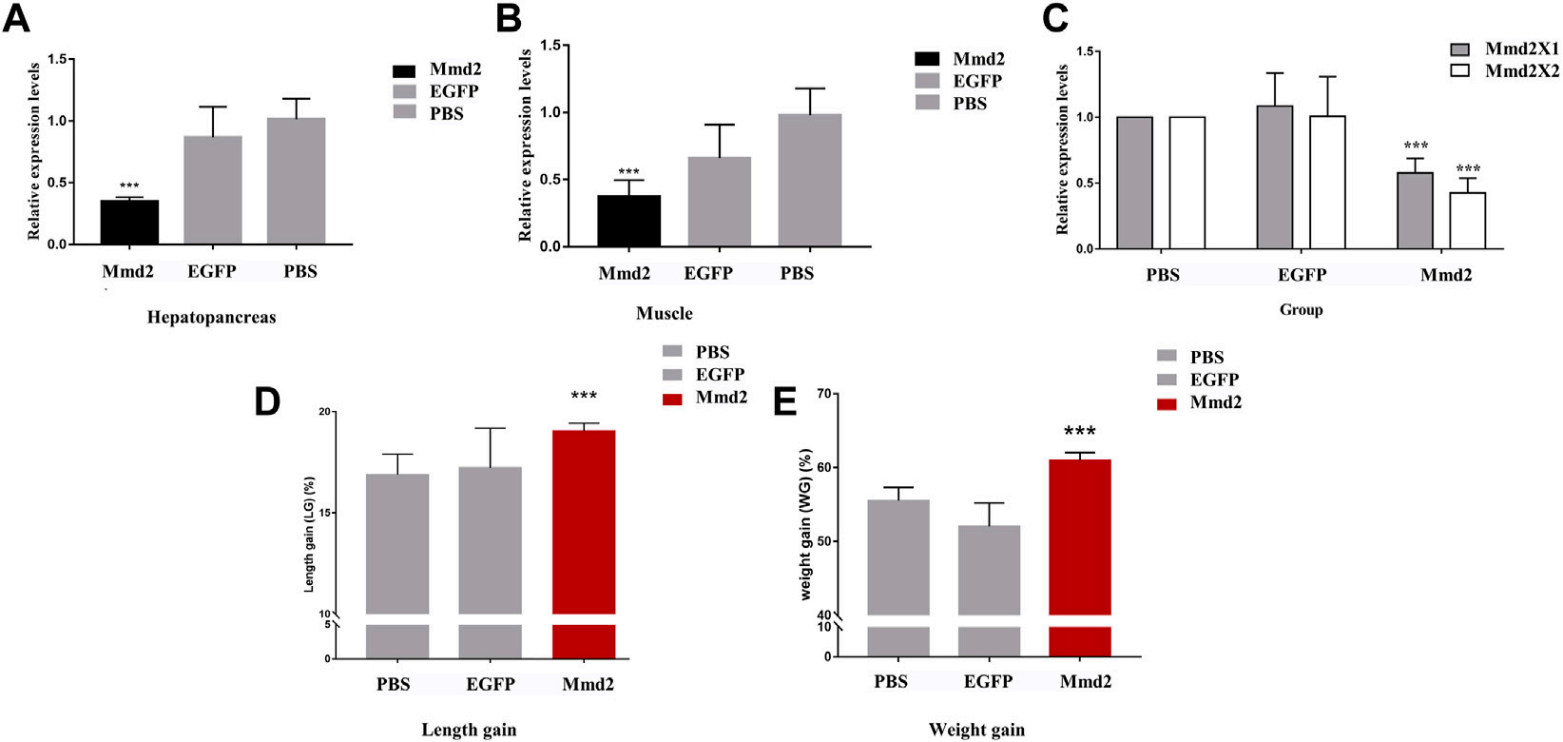
The results of LvMmd2 gene RNA interference in *Litopenaeus vannamei*. **(A)** Relative expression level of *LvMmd2* after RNAi in hepatopancreas. **(B)** Relative expression level of *LvMmd2* after RNAi in muscle. **(C)** Relative expression level of *LvMmd2X1* and *LvMmd2X2* after RNAi in hepatopancreas. **(D)** The increase in body weight after *LvMmd2* RNAi. **(E)** The increase in body length after *LvMmd2* RNAi. PBS group and EGFP group were used as the control group. The expression of target genes was detected by qRT-PCR and normalized to the 18S rRNA gene as the internal reference. These results were based on three independent biological replications and are shown as mean values ±SD. Significant differences in the gene expression levels between the three treatments are shown as **p < 0.01.

RNA interference primer was designed at the homologous region of *LvMmd2X1* and *LvMmd2X2* to synthesize dsRNA and knock down the whole *LvMmd2* gene, (Supplementary Figure S1A). Compared with the dsEGFP and PBS groups, the expression of *LvMmd2* gene was significantly reduced when 2 µg dsMmd2 was injected (Supplementary Figure S1B). Hence, the optimal interference dosage 2 µg was used for every individual. After 2 weeks formal RNA interference experiment, the expression of LvMmd2 gene in the hepatopancreas and muscles were significantly decreased by dsMmd2 (Figures 5A,B). The interference efficiency of *LvMmd2X1* and *LvMmd2X2* was detected by the specific primers. It was found that both they were knocked down, and the knockdown efficiency of *LvMmd2X2* was higher than *LvMmd2X1* (Figure 5C).

After the RNA interference experiment, statistics of growth traits showed that the body weight and length gain of the dsMmd2 group were markedly higher than those of the dsEGFP and PBS groups, which was consistent with the relative expression levels of *LvMmd2* in different growing rate populations (Figures 5D,E).

### 3.5 Comparative transcriptome analysis after *LvMmd2* knocked down

#### 3.5.1 RNA-sequencing data

To estimate the changes in gene expression induced by the *LvMmd2* knockdown, we compared the transcriptomes of hepatopancreas tissues with and without dsMmd2 injection. In this study, 284.07 M raw reads were obtained from six sequencing libraries. The effective data amount was distributed in 6.21–7.44 Gb in each library, the Q30 varied from 92.77% to 93.8% (Supplementary Table S4). All raw reads had been deposited in the NCBI Sequence Read Archive (SRA) website (accession number PRJNA918508). Compared the obtained clean reads with the genome database of *L. vannamei*, as shown in Supplementary Table S5, more than 90% of the clean reads could be matched to the genome database.

#### 3.5.2 Identification of DEGs

**FIGURE 6:**
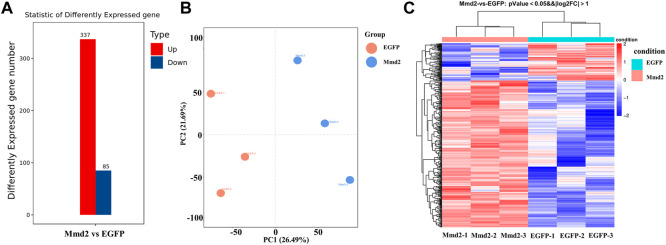
DEGs of hepatopancreas in *Litopenaeus vannamei* after *LvMmd2* RNAi. **(A)** The number of DEGs among hepatopancreas. **(B)** The PCA of all DEGs in hepatopancreas. **(C)** The heat map of DEGs among three parallel hepatopancreas. Abscissa for |log2FoldChange| > 1, Ordinate for *p*-value <0.05.

Based on sequencing data, the expression level of each gene was calculated. According to transcriptome data, DEGs were found, including 85 downregulated genes and 337 up-regulated genes (Figure 6A). In order to verify the iterancy of each group and the validity of the grouping of the experimental and control groups, we performed principal component analysis (PCA) (Figure 6B). The result indicated that there was good repeatability in each group, and there was a distance between *LvMmd2* RNAi group and control group, which indicated they were clustered together separately. In order to further investigate the expression patterns of the DEGs selected from the dsMmd2 and control groups, we conducted cluster analysis on these DEGs (Figure 6C).

The analysis of transcriptome showed that there were significant differences in many genes between the RNAi group and the control group (Table 3). Among them, some essential components of muscle cytoskeleton and some genes involved in muscle development or differentiation were only upregulated in *LvMmd2* RNAi group, such as *actin*, *myosin* and epidermal growth factor receptor (*EGFR*). There are also some genes related to improving immunity and stress, which were also only upregulated in *LvMmd2* RNAi group, such as C-type lectin family genes, mannose receptor (*MRC2*), superoxide dismutase (*SOD*), etc. However, we also found some genes involved in signal transduction, protein digestion, absorption and transport, which were significantly upregulated or down-regulated after *LvMmd2* interference. In addition, other down-regulated genes were found in the *LvMmd2* dsRNA group, such as Ras superfamily genes and genes involved in glucose metabolism.

**TABLE 3:** Some DEGs in hepatopancreas after *LvMmd2* RNAi.

Gene	Annotation	Log2(fold change)	Regulation	Possible functions
LOC113803355	actin-like	4.545	Up	essential component of the muscle cytoskeleton
LOC113815142	actin-like	3.291563252	Up	
LOC113805465	myosin regulatory light chain 2-like	2.392415647	Up	
LOC113823552	myosin regulatory light chain 2-like	2.351348161	Up	
LOC113822686	myosin light chain alkali-like	1.0525679	Up	
LOC113824982	collagen alpha-3(IX) chain-like	1.35185696	Up	
LOC113813068	collagen alpha-1(I) chain-like	1.198428831	Up	
LOC113827072	Collagen alpha-1(XI) chain	1.167199758	Up	
LOC113807987	collagen alpha-1(I) chain-like	1.0131205	Up	
LOC113815446	chondroitin proteoglycan-2-like	1.187366726	Up	
LOC113815782	chondroitin proteoglycan 2-like	1.018277715	Up	
LOC113823280	Epidermal growth factor receptor	2.008971472	Up	
LOC113815384	Peritrophin-1	1.457802192	Up	structural protein related genes
LOC113816928	Peritrophin-1	1.09799355	Up	
LOC113824427	trypsin-like	1.451603354	Up	protein digestion, absorption and transport
LOC113814621	solute carrier family 22 member 6-like	1.561611039	Up	
LOC113810126	solute carrier family 2, facilitated glucose transporter member 1-like	1.384393286	Up	
LOC113818126	glutamate receptor 3-like	−5.566253426	Down	
LOC113809326	proteasome subunit alpha type-7-like	−4.896032637	Down	
LOC113813297	protein transport protein Sec61 subunit alpha-like 1	−1.587305241	Down	
LOC113822227	probable chitinase 10	−2.99304761	Down	
LOC113813817	apolipoprotein D-like	−1.043054937	Down	
LOC113810219	crustacyanin-A2 subunit-like	−1.014815483	Down	
LOC113812656	sarcoplasmic calcium-binding protein, beta chain-like	4.11463727	Up	signal transduction
LOC113810364	Sarcoplasmic calcium-binding protein, beta chain	2.627430308	Up	
LOC113814611	Sarcoplasmic calcium-binding protein, alpha-B and -A chains	2.234974484	Up	
LOC113818538	calcium-activated chloride channel regulator 4A-like	1.478338819	Up	
LOC113829602	Calcium-activated chloride channel regulator 2	1.390405857	Up	
LOC113817417	1-phosphatidylinositol 4,5-bisphosphate phosphodiesterase beta-4	1.762627199	Up	
LOC113823850	TRPM8 channel-associated factor 3-like	−1.114459946	Down	
LOC113829337	putative protein TPRXL	−1.444823194	Down	
LOC113826226	Rho GTPase-activating protein 27 like	−6.041117418	Down	
LOC113809417	ras-related protein Rap-1b-like	−0.315803881	Down	
LOC113824295	leukocyte elastase inhibitor B-like	1.236358842	Up	immune related genes
LOC113825092	C-type lectin domain family 17, member A-like	4.032764854	Up	
LOC113804438	C-type mannose receptor 2	2.82362366	Up	
LOC113803834	techylectin-5B-like	1.177860197	Up	
LOC113807384	superoxide dismutase [Cu-Zn]-like	1.743252232	Up	
LOC113806394	superoxide dismutase [Cu-Zn]-like	1.481862801	Up	
LOC113824103	putative glucosylceramidase 3	−1.11065931	Down	sugar synthesis and breakdown
LOC113824094	glucosylceramidase-like	−1.071736219	Down	
LOC113809777	glycogen debranching enzyme-like	−1.113569457	Down	
LOC113828355	beta-glucuronidase-like	−1.323795209	Down	
LOC113816884	beta-1,4-glucuronyltransferase 1-like	−1.297255518	Down	

#### 3.5.3 GO enrichment analysis

**FIGURE 7:**
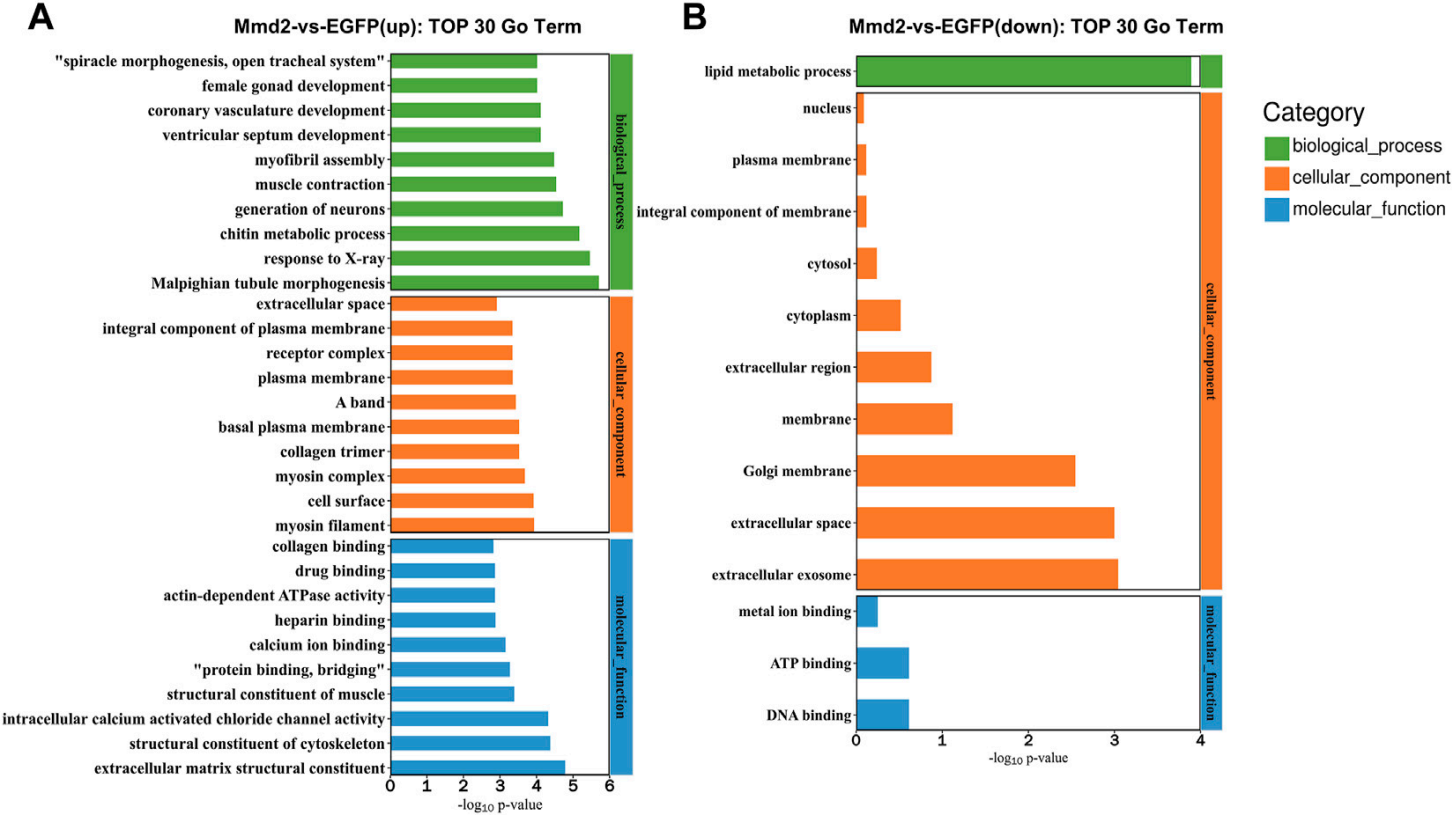
Top 30 GO terms of DEGs. **(A)** Top 30 GO terms of biological functions of upregulated. **(B)** Top 30 GO terms of biological functions of downregulated.

The DEGs were analyzed for functional annotation in the GO database. The results showed that DEGs were annotated into biological process, molecular function and cellular component. The 337 up-regulated DEGs were significantly concentrated to 48 GO terms and the 85 down-regulated DEGs were significantly concentrated to 41 GO terms (Supplementary Figure S2). We extracted the top 30 GO items of the DEGs as shown in Figure 7. The top 30 GO items of up-regulated DEGs mainly focus on chitin metabolic process, generation of neurons, muscle contraction, extracellular matrix structural constituent, structural constituent of cytoskeleton, and intracellular calcium activated chloride channel activity. The top 30 GO terms of down-regulated DEGs are lipid metabolic process, extracellular exosome, extracellular space, and Golgi membrane, etc (Supplementary Tables S6, S7).

#### 3.5.4 KEGG enrichment analysis

**FIGURE 8:**
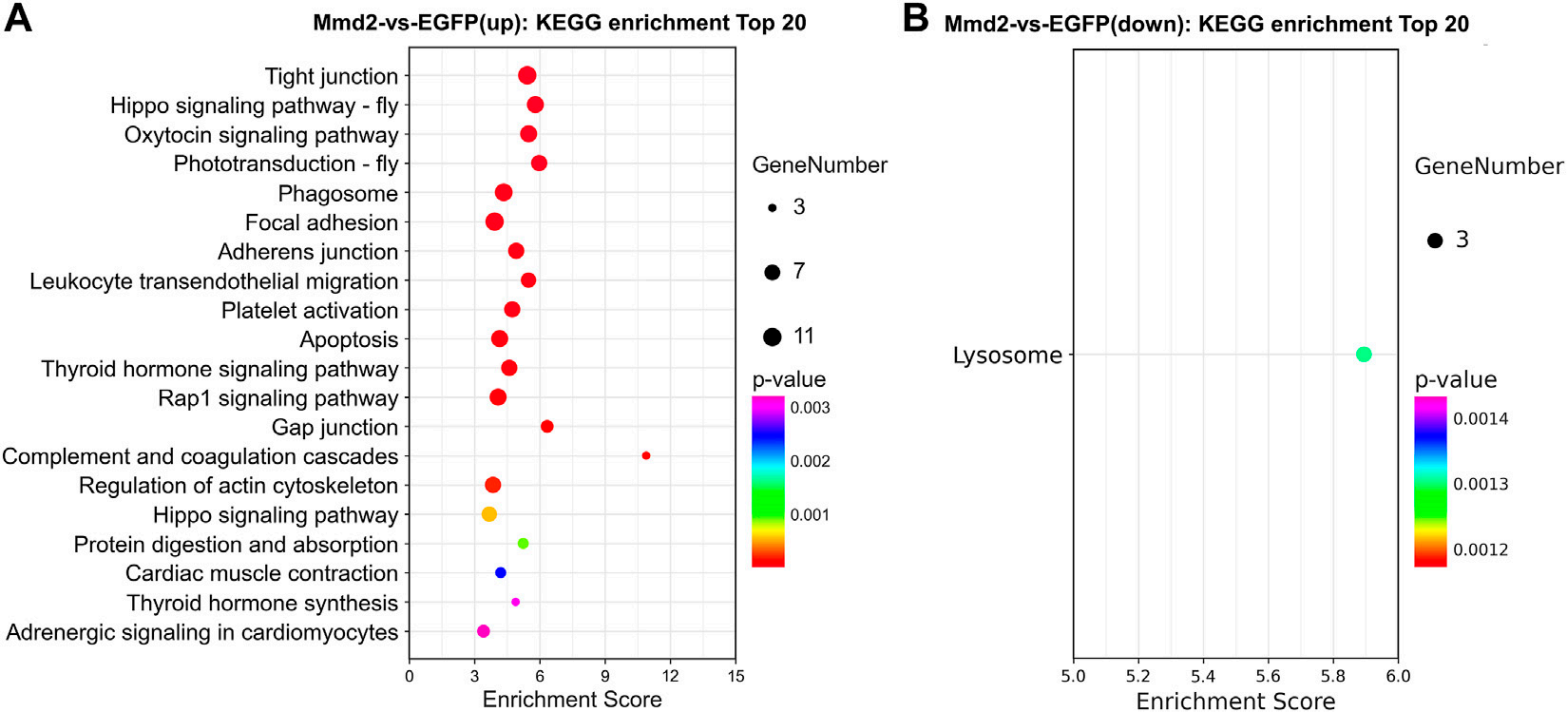
The pathways of KEGG enrichment analysis in the hepatopancreas of *Litopenaeus vannamei* after *LvMmd2* RNAi. **(A)** KEGG enrichment pathway analysis of upregulation gene. **(B)** KEGG enrichment pathway analysis of downregulation gene.

The signal pathways enriched by the DEGs were analyzed in the KEGG database. KEGG enrichment analysis showed upregulated DEGs were markedly enriched in 20 pathways, while the down-regulated DEGs were markedly enriched in only one pathway (ListHits>3, *p*-value <0.05) (Figure 8). The top enriched pathways included a lot of development related pathways and related to immune defense and metabolic processes (Supplementary Tables S8, S9). Among them, The signaling pathways significantly enriched by up-regulated DEGs include regulation of actin cytoskeleton, protein digestion and absorption, oxytocin signaling pathway, Hippo signaling pathway, adrenergic signaling in cardiomyocytes, cardiac muscle contraction, thyroid hormone signaling pathway, Wnt signaling pathway and Rap1 signaling pathway. The down-regulated DEGs enriched in lysosome signaling pathway.

#### 3.5.5 Verification of the RNA-Seq results by qPCR

**FIGURE 9:**
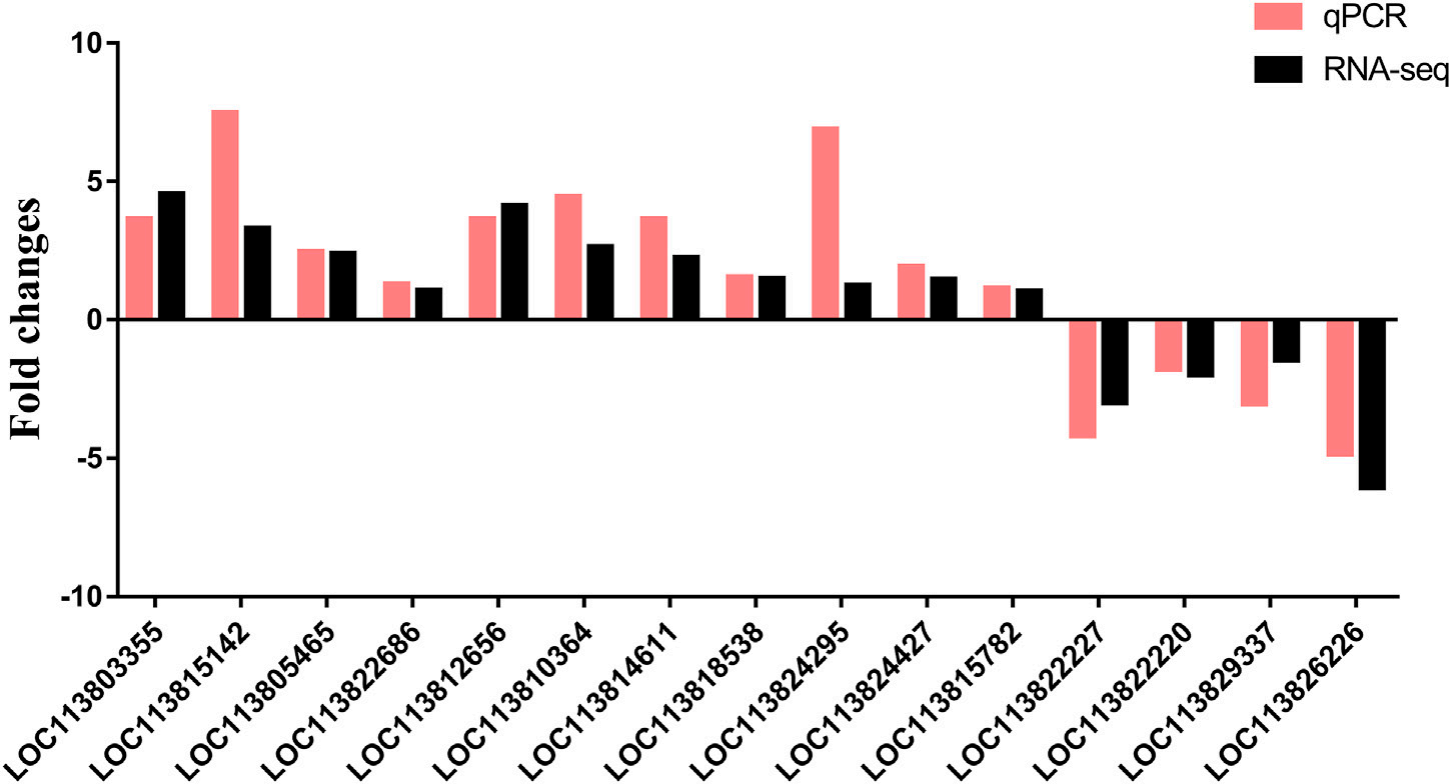
Verification of differentially expressed genes by qPCR.

To verify these DEGs found by RNA-Seq, the relative expression levels of 15 genes in hepatopancreas were detected by RT-qPCR (Figure 9). A total of fifteen DEGs were randomly selected from transcriptome sequencing data for the gene expression results verification. As showed in Figure 9, the expression patterns of these fifteen DEGs were consistent with the RNA sequencing, which conform the expression changes of these genes and the reliability and accuracy of the RNA-Seq results.

## 4 Discussion

### 4.1 Gene structure and expression of *LvMmd2*

In our previous studies, through GWAS analysis, a growth-related gene *LvMmd2* was identified from the *L. vannamei* genome (Wang et al., 2020). In this study, the gene structure, expression characteristics and function of *LvMmd2* were analyzed more deeply. Two alternative splice forms, *LvMmd2X1* and *LvMmd2X2* were detected, both of which are acidic proteins and have a conserved Pfam-HLYIII domain. Among the two alternative splice forms, *LvMmd2X1* is the same as the growth-related gene reported previously (Wang et al., 2020), the *LvMmd2X2* has an extra transmembrane region compared with *LvMmd2X1*, and the expression level of *LvMmd2X2* is higher than *LvMmd2X1* universally. The two alternative splice forms were expressed very differently in different adult tissues, different early development stages, different molting stages, and different pathogen infections, they may perform different functions in subtle aspects, however, the differences of their specific functions are still unclear.

In previous studies, *LvMmd2* was identified as a gene encoding PAQR10 (Wang et al., 2020). The characteristic of the PAQR family is an ancient 7-TM structural protein, and exists eukaryotes, bacteria, and fungi (Tang et al., 2005). It is reported that invariant residues of PAQR protein are found mostly in the intracellular loops and the intracellular face of the transmembrane structure domains (Tang et al., 2005). Interestingly, consistent with the reported characteristics, The predicted transmembrane domain sites in the TM domain of *LvMmd2* indicated that invariant residues were also mainly found in the intracellular loops and the surface of the transmembrane structure domains (Figure 2). This indicated that similar to GPCRs, the conserved features may be necessary for cell signal transduction. For example, the sequence of rhodopsin-type GPCRs is relatively low, but they maintain a seven transmembrane structure with highly conserved residues (Gether, 2000). By sequence alignment, the same domain of PAQR10 or Mmd2 protein was found in different species, and highly conserved invariant residues were found in intracellular circulation or intracellular TM domain, suggesting a high homology of the Mmd2 gene between invertebrates and vertebrates. We found that the topology of Mmd2 was similar to GPCR family members as both of them contain multiple transmembrane motifs. The difference is that the former loses TM1 and TM6 in most species during evolution suggests that TM1 and TM6 may not be the main sites for PAQR10. The structure of the members of progesterone membrane protein related receptors subgroup is similar to GPCRs, and its N-terminal is extracellular (Zhu et al., 2003; Thomas, 2008). However, the N-terminal of the members of adiponectin related receptors subgroup is intracellular, the C-terminal is extracellular, and the protein structure is significantly different from traditional GPCRs, which is unique to members of the PAQR family (Vassilatis et al., 2003; Yamauchi et al., 2003). Regarding hemolysin related receptors, the N-terminal of *LvMmdX1* is intracellular, the N-terminal of *LvMmd2X2* is extracellular, and the C-terminal of both are extracellular.

### 4.2 Constitution of the shrimp PAQR family

To this day, there are 11 members of the PAQR family found in humans, they were divided into three main subgroups: adiponectin related receptors, including PAQR1 (AdipoR1), PAQR2 (AdipoR2), PAQR3 and PAQR4; progesterone membrane protein related receptors, including PAQR5 (mPRγ), PAQR6, PAQR7 (mPRα), PAQR8 (mPRβ), PAQR9; and hemolysin related receptors, including PAQR10, PAQR11 (Fernandes et al., 2005; Tang et al., 2005). In addition, YOL002c and zh1p ∼ Izh4p exist in yeast, they are the members of PAQR family but have not been identified in mammals (Fernandes et al., 2005). The most divergent members in the PAQR family are PAQR 10 and PAQR11, they have high sequence similarity with the bacterial hemolysin III type proteins compared to the other PAQR members, suggesting that they are the most likely descendants of the original PAQR family (Tang et al., 2005). In *L. vannamei*, besides the hemolysin related receptor PAQR10 (*Mmd2*) gene, other members of the PAQR family sequences have also been found, including AdipoR1, PAQR3, and PAQR9. The phylogenetic tree of PAQR family of *L. vannamei* and other species showed that the PAQR family was indeed divided into three categories (Supplementary Table S10; Supplementary Figure 3). In shrimp, the members of the PAQR family do not seem to be as many as those of mammals, which may indicate that these members still maintain an ancient state. As for the function of these PAQR genes, no research has been conducted in crustacean so far.

### 4.3 The effect of *LvMmd2* on growth

In vertebrates, most studies on Mmd2 gene focus on regulating apoptosis signaling pathway (Góñez et al., 2008; Jin et al., 2012a; Jin et al., 2012b). According to the expression profile of different tissues in adult shrimp, LvMmd2 gene was highly expressed in the eye stalk, ovary and hepatopancreas. In crustaceans, the neuroendocrine organ, X-organ–sinus-gland complex is located in the eye stalk and regulates the molting, growth, reproduction, and many physiological and metabolic processes (Choy, 1987; Chen et al., 2018), the hepatopancreas is mainly involved in the digestion and absorption of nutrients, and also a key organ for immune regulation in shrimp (Vogt et al., 1989; Huang et al., 2020). In this study, the increase of body length and body weight in dsMmd2 group was significantly higher than control groups, this results confirmed our previous research findings, *LvMmd2* is a gene that plays an important role in shrimp growth traits (Wang et al., 2020). Further gene interaction prediction also indicated that *LvMmd2* might interact with genes (*ANO5a*, *ANO5b*, and *DYSF*) related to growth and development, these genes have been discovered by researchers to play an important role in mammal muscle development (Liu et al., 1998; Hicks et al., 2010; Wang et al., 2020). Previously, two growth-related genes, *PKC-delta* and *Rap-2a*, were discovered in *L. vannamei* (Yu et al., 2019). Researchers have found that Mmd2 is involved in the formation of late endosomes/lysosomes, and this process can be blocked by activation of protein kinase C-dependent pathways (Bräuer et al., 2004). Besides, Rap-2a, as a member of the Ras superfamily, which may play an important role in cell proliferation and differentiation (Jin et al., 2015), and Mmd2 can mediate the Ras signaling in mammalian Golgi apparatus. So, whether these three genes are interrelated and jointly regulate growth deserves further in-depth research.

### 4.4 Possible functions of *LvMmd2*

*LvMmd2* gene is widely expressed in various tissues of *Litopenaeus vannmei* in different alternative splicing forms, indicating that its biological functions are very complex. Interestingly, *Mmd2* has previously been identified expression in mouse developing testis, but not in ovary (Jameson et al., 2012; Zhao et al., 2018; Zhao et al., 2022), however, *LvMmd2* was indeed more highly expressed in ovary than testis in shrimp, which suggest that Mmd2 has great functional differences between mammal and crustacean. In vertebrates, studies have shown that Mmd2 can control the proliferation of glial precursors within the spinal cord, which controlled by SOX9 transcription factors (Kang et al., 2012), however, it has been noted that SOX9 has the opposite function of promoting and inhibiting proliferation, which showed that Mmd2 exhibits functional diversity according to different environments (Shi et al., 2015).

Through transcriptome analysis after *LvMmd2* knockdown, many of differentially expressed genes were found associated with growth. A number of muscle protein genes, including *myosin*, *actin*, *collagen alpha-1*, sarcoplasmic calcium-binding proteins (*SCP*s), were found strongly up-regulated exclusively in *LvMmd2* RNAi group. Actin, Myosin, and Collagen are the structural proteins of muscle and other body tissues, Sarcoplasmic calcium-binding protein and Calcium-activated chloride channel can participate in calcium transport and thus also promote calcium absorption (E. Rohrback et al., 2015). In invertebrates, up-regulation of genes related to muscle composition leads to greater locomotion or swimming ability, enabling rapid hunting or avoidance of predation. Additionally, some genes that promote digestion and absorption were significantly upregulated in *LvMmd2* RNAi group, such as *peritrophin*, *trypsin*, *SLC*s, etc. Peritrophin is composed mainly of chitin and proteins that protect epithelial cells in the midgut and aid in food digestion and absorption. Trypsin is primarily involved in food digestion, hydrolysis, and activation of zymogens (Shi et al., 2015). Solute carrier proteins (*SLC*s) are mainly responsible for transporting various macromolecular substances such as lipids, amino acids, sugars, neurotransmitters, and drugs between biofilms (Saikrithi et al., 2020).

Besides, some immune-related genes were found upregulated only after *LvMmd2* RNAi as well, suggesting that *LvMmd2* also affects the immunity of the shrimp. The expression of C-type lectin domain family 17 member A-like (*CLEC17A*) and C-Type mannose receptor 2 (*MRC2*) was significantly upregulated, and *Techylectin 5B*, a novel lectin tachylectin-related protein gene identified in species ranging from ancient sponges to bony fish, which have been found to play an important role in innate immunity, was also up-regulated. Superoxide dismutase (*SOD*) was significantly up-regulated which has a key role in shrimp for the prevention of oxidative damage development and induction of apoptosis (González-Ruiz et al., 2021). These DEGs may promote shrimp growth by increasing muscle production and improving the shrimp’s digestion and absorption, and immune system.

The enrichment of DEGs on biological process (GO terms) provided a considerable perspective for understanding the biological changes after *LvMmd2* RNAi. Top 30 GO terms of biological functions of up-regulated associated with muscle and skeletal development including muscle contraction, structural constituent of cytoskeleton, myofibril assembly, myosin filament, collagen trimer, structural constituent of muscle, collagen binding. In addition, there are some GO biological processes terms involved with metabolism process including chitin metabolic process, however, down-regulated genes were significantly enriched in lipid metabolism and membrane changes.

The KEGG pathway enrichment analysis identified at least eight growth-related signaling pathways in the up-regulated DEGs, including the thyroid hormone signaling pathway, regulation of actin cytoskeleton, cardiac muscle contraction, protein digestion and absorption, Rap1 signaling pathway, Wnt signaling pathway, Hippo signaling pathway and thyroid hormone synthesis. The Hippo pathway is highly conserved and limits organ size by phosphorylating, and inhibiting the transcription co-activators YAP/TAZ in mammals and Yki in *Drosophila*, which are all key regulators of cell proliferation and apoptosis (Wang et al., 2016). In this study, RNA-Seq data showed that *Lft*, *Egr* and *F-actin* were upregulated and significantly enriched in Hippo pathway. Their upregulated expression may inhibit the expression of *Warts*, *Hippo* and *Salvador*. YAP and TAZ are transported to the nucleus and may act as transcriptional coactivators of TEADs to promote transcription, thus promote the growth of tissues and organs in shrimp. Thyroid hormone (Th) contains thyroidine precursors (T4) and active form triiodothyronine (T3), which play an important role in the growth and metabolism of vertebrates (Huang et al., 2015), they are also critical for promoting larval growth and induction of metamorphosis in both invertebrates and vertebrates (Alinezhad et al., 2020). We found the upregulated genes enriched in the thyroid hormone pathway downstream, they conduct the thyroid hormone signal outside the nucleus (PLC/PKC→MAPK/ERK). In terms of thyroid hormone pathway, there were two up-regulated genes, phospholipase C (*PLC*) and *actin*, in *LvMmd2* RNAi group. The significantly up-regulated *PLC* might promote the hydrolysis of phosphatidylinositol 4, 5-diphosphate (PIP2) to inositol 1, 4, 5-triphosphate (IP3) and diacylglycerol (DAG) acting on PKC, which in turn enhanced ERK phosphorylation. Compared with control group, the up-regulated genes in *LvMmd2* RNAi group were enriched into Wnt pathway, and the upregulated expression of *PLC* might enhance the Wnt pathway and promoted the cell proliferation regulation in shrimp. The activity changes of Wnt signaling can affect the growth and immunity of *L. vannamei* probably by regulating metabolic and immune process (Zhang et al., 2021). Members of the Wnt family have been shown to modulate convergent extension movements, and cell differentiation (Kühl et al., 2001). In transcriptome data, the expression of receptor tyrosine kinase (*RPTK*), a high-affinity cell surface receptor of numerous polypeptide growth factors, cytokines and hormones, was significantly upregulated to activate the PLC gene. At the same time, other enrichment signal pathways, such as the Rap1 signaling pathway, regulation of actin cytoskeleton, longevity regulating pathway, protein digestion and absorption, glycosaminoglycan biosynthesis and cardiac muscle contraction are also significant upregulated in *LvMmd2* RNAi group. These results can be concluded that *LvMmd2* is a growth inhibiting gene, after its RNA interference, growth, metabolism and immune related signaling pathways were significantly activated.

## 5 Conclusion

In this study, the *Mmd2* gene was further identified, characterized and function analyzed using the genome, transcriptome and RNA interference in *L. vannamei*. The results showed that LvMmd2 (PAQR10) gene had the same highly conserved domain and homologous sequence, it was widely expressed with two alternative splicing forms, might have multiple functions. RNA interference experiment showed that *LvMmd2* may participate in growth as an inhibitory gene in *L. vannamei*. Various muscle-related genes, protein synthesis and decomposition genes, and immune-related genes were significantly upregulated in the *LvMmd2* RNAi groups, suggesting a potential contribution of *LvMmd2* genes in weight gain and rapid growth. Enrichment analysis, as well as further investigation into the DEGs, revealed the main changes after *LvMmd2* knockdown were in growth, metabolism, and immune-related biological processes. Our findings provide a valuable basis for further research and utilization Mmd2 gene in shrimp and crustacean.

## Data Availability

The data presented in the study are deposited in the NCBI Sequence Read Archive (https://www.ncbi.nlm.nih.gov/sra), accession numbers: PRJNA918508, SRR22981843-SRR22981848.
