# Supplementary material for: Structure and function analyses of the Mmd2 gene in pacific white shrimp Litopenaeus vannamei
Source: Front Genet. 2023 Jul 7;14:1151193. doi: 10.3389/fgene.2023.1151193 (PMC10361620; doi:10.3389/fgene.2023.1151193)
Supplement: Supplementary file 1 [file DataSheet1.pdf]

Supplementary Material

Structure and Function Analyses of the Mmd2 Gene in Pacific White Shrimp *Litopenaeus vannamei*

Shuqing Si[1,2], Xiaojun Zhang[1,2,3,4,5*], Yang Yu[1,3,4,5], Xiaoxi Zhang[1,3,4], Xiaoyun Zhong[1,5], Jianbo Yuan[1,3,4,5], Song Yang[2], Fuhua Li[1,3,4,5]

[1]CAS and Shandong Province Key Laboratory of Experimental Marine Biology, Institute of Oceanology, Chinese Academy of Sciences, Qingdao 266071, China

[2]School of Life and Sciences, Qingdao Agricultural University, Qingdao 266109, China

[3]Laboratory for Marine Biology and Biotechnology, Qingdao National Laboratory for Marine Science and Technology, Qingdao 266237, China

[4]Center for Ocean Mega-Science, Chinese Academy of Sciences, Qingdao 266071, China

[5]University of Chinese Academy of Sciences, Beijing, China

*** Correspondence:**
Corresponding Author
e-mail address: xjzhang@qdio.ac.cn(Xj Zhang)

1 Supplementary Tables

Table S1 The sequence information for Mmd2 phylogenetic analysis

Sequence ID	Length/aa	annotation	species
XP_027234996.1	217	monocyte to macrophage differentiation factor 2-like	*Litopenaeus vannamei*

c83205_g1	274	monocyte to macrophage differentiation factor 2-like	*Litopenaeus vannamei*
XP_037786106.1	274	monocyte to macrophage differentiation factor 2-like isoform X2	*Penaeus monodon*
XP_042889243.1	274	Monocyte to macrophage differentiation factor 2	*Marsupenaeus japonicus*
XP_047492922.1	274	Monocyte to macrophage differentiation factor 2	*Fenneropenaeus chinensis*
XP_042243198.1	284	Monocyte to macrophage differentiation factor 2	*Homarus americanus*
XP_045593874.1	266	Monocyte to macrophage differentiation factor 2	*Procambarus clarkii*
RXG69871.1	253	Monocyte to macrophage differentiation factor 2	*Armadillidium vulgare*
XP_045137938.1	278	Monocyte to macrophage differentiation factor 2	*Portunus trituberculatus*
KAG0725822.1	303	Monocyte to macrophage differentiation factor 2	*Chionoecetes opilio*
MCL4129961.1	210	Monocyte to macrophage differentiation factor 2	*Idotea baltica*
XP_037074221.1	237	Monocyte to macrophage differentiation factor 2	*Pollicipes pollicipes*
XP_021925216.1	267	Monocyte to macrophage differentiation factor 2	*Zootermopsis nevadensis*
XP_015512793.1	277	Monocyte to macrophage differentiation factor 2	*Neodiprion lecontei*
XP_022202893.2	260	Monocyte to macrophage differentiation factor 2	*Nilaparvata lugens*

XP_046987717.1	254	Monocyte to macrophage differentiation factor 2	*Schistocerca americana*
XP_018964236.1	235	monocyte to macrophage differentiation factor 2-like isoform X1	*Cyprinus carpio*
NP_001167529.1	243	monocyte to macrophage differentiation factor 2a	*Danio rerio*
AAR08376.1	246	Monocyte to macrophage differentiation factor 2	*Homo sapiens*
NP_780426.1	242	Monocyte to macrophage differentiation factor 2	*Mus musculus*
XP_001630086.1	229	monocyte to macrophage differentiation factor 2	*Nematostella vectensis*
NP_014641.2	317	PAQR-type receptor YOL002c	*Saccharomyces cerevisiae S288C*
CAF1561142.1	316	Izh1p	*Saccharomyces cerevisiae PE-2*
CAF1605453.1	317	Izh2p	*Saccharomyces cerevisiae PE-2*
NP_013123.1	543	Izh3p	*Saccharomyces cerevisiae PE-2*
NP_014540.1	312	Izh4p	*Saccharomyces cerevisiae PE-2*
MBS5324544.1	228	hemolysin III family protein	*Lachnospiraceae bacterium*
MBS6159742.1	220	hemolysin III family protein	*Firmicutes bacterium*
ASU30563.1	220	hemolysin	*Blautia pseudococcoides*

Table S2 The primers designed for dsRNA compound of *LvEGFP* and *LvMmd2*

Primer ID		Primer sequence (5'-3')	Fragment length (bp)	Ta (°C)
PrimerdsEGFP	dsEGFP-F	TAATACGACTCACTATAGGGCAGTGCTTCAGCCGCTACCC	289	65
	dsEGFP-R	TAATACGACTCACTATAGGGAGTTCACCTTGATGCCGTTCTT		
PrimerdsMmd2	dsMmd2-F	TAATACGACTCACTATAGGGCCTTCCTCTCACTCAGTTATTTG	507	65
	dsMmd2-R	TAATACGACTCACTATAGGGCCATCCAGCTTAAAGAAGATCAC		

Table S3 The primers designed for Real-Time Quantitative PCR experiments

Primer ID		Primer sequence (5'-3')	Fragment length (bp)	Ta (°C)
Primer-Mmd2	RT-Mmd2-F	GCAGCCTTTATCCATTACTATGC	202	57
	RT-Mmd2-R	GCAGCCTTTATCCATTACTATGC		
Primer-	RT-LOC113808843-F	AAGGTTTTTGCGTCTCTTTGTC	189	57

LOC113808843	RT-LOC113808843-R	TACAGAAATCACCACGAACACA		
Primer-LOC113803303	RT-LOC113803303-F	TTGATGAGCCTTGGCAAGTTAG	207	57
	RT-LOC113803303-R	TTCACTTGAGGATCTTGCGAAC		
Primer-LOC113807250	RT-LOC113807250-F	GTTGTCCAAGTGTATCACGGG	168	57
	RT-LOC113807250-R	AATCGGTTTGTCCATCTTGGC		
Primer-LOC113819693	RT-LOC113819693-F	GTTCCCCTCTCGTCCAGATAAT	212	57
	RT-LOC113819693-R	TAACATCTCCAAGGACGTCGAT		
Primer-LOC113820150	RT-LOC113820150-F	AGGAGCAAACTGTCTAGGAACA	197	58
	RT-LOC113820150-R	GACGATAATGGGTAGCCATTCG		
Primer-LOC113823280	RT-LOC113823280-F	ACTTGGAAGTGACTCACTGTCT	187	58
	RT-LOC113823280-R	CTATGGGCATGTCGCTTATCTG		
Primer-LOC113826331	RT-LOC113826331-F	CCTCTCAGTCCAACTACATCGA	195	58
	RT-LOC113826331-R	TCCTTGTTGAACTGAGACTCCA		
Primer-	RT-LOC113827350-F	GCGACACAATCAAGGAGAATGA	202	58

LOC113827350	RT-LOC113827350-R	TCTTGATGTTCTGCTGTTTGCT		
Primer-LOC113830072	RT-LOC113830072-F	GAATTCCCCTTCTGGTGGAATG	207	58
	RT-LOC113830072-R	TTGTTCCACTGGAGTTCACCTA		
Primer-LOC113805950	RT-LOC113805950-F	ATCTCCCCTGCTAAGAAGACTG	204	58
	RT-LOC113805950-R	GCAGACTATTTTCCGAGGCTTT		
Primer-LOC113807835	RT-LOC113807835-F	ACAGGAAACCAAATGCGAACA	249	58
	RT-LOC113807835-R	TCGAAGAAGAGTCCTCTACGC		
Primer-LOC113823257	RT-LOC113823257-F	CTTCTGCCATGTCGTCAGATG	225	57
	RT-LOC113823257-R	CGCTCTCTTTCTTGAGACACG		
Primer-LOC113806042	RT-LOC113806042-F	GTGGTTGACTCTGAAGATCTGC	220	59
	RT-LOC113806042-R	AGGTTCTTGGAGAAGTTCTCGT		
Primer-LOC113827868	RT-LOC113827868-F	GCGAATCTGGCTCTATGTTCTC	186	58
	RT-LOC113827868-R	TGGAACTGCTTCTTCAGACTCT		
Primer-	RT-LOC113802836-F	CATAACCAATGCCACGTACCTT	192	58

LOC113802836	RT-LOC113802836-R	GTCCGTGGGTCATTAATACGTC		
Primer-LOC113807834	RT-LOC113807834-F	CGAAATTCCAATCACGAGGTCA	208	56
	RT-LOC113807834-R	GTTCTCAGTCGCTCTTGTCTTC		
Primer-LOC113823256	RT-LOC113823256-F	CTTCTGCCATGTCGTCAGATG	171	57
	RT-LOC113823256-R	AACACCTTCCTTGTTGCAGAC		
Primer-LOC113820865	RT-LOC113820865-F	CTCCCATCCAAACTGCTGATG	189	57
	RT-LOC113817999-R	TTGCGGACGTGATGGTAATTC		
Primer-LOC113817999	RT-LOC113817999-F	AGCACGGCTTCCACATTCA	207	57
	RT-LOC113823256-R	TCACCTCGTCGTCCAGGAT		
Primer-LOC113809633	RT-LOC113809633-F	ACTGGTACCAGAATCACCCTC	168	57
	RT-LOC113809633-R	GACGTAAACTTCTCTGCGACC		
Primer-LOC113800820	RT-LOC113800820-F	CATCGCTGCCATCAAGACAA	212	57
	RT-LOC113800820-R	GGTCGTGTTGGAGATCATGC		
Primer-	RT-LOC113806076-F	TTGCAGTAAACATGGTGCCC	197	58

LOC113806076	RT-LOC113806076-R	TGGACACTCTGCCTCTGAAG		
Primer-LOC113809896	RT-LOC113809896-F	CAAGCACGAGAAATCCGAGG	187	58
	RT-LOC113809896-R	AGCGAGGTTTTTCTCCGGTA		
Primer-LOC113803355	RT-LOC113803355-F	GATGACTCAGATCATGTTCGAGT	195	58
	RT-LOC113803355-R	AGGCAATAACCTTCGTATACAGG		
Primer-LOC113815142	RT-LOC113815142-F	GATGACTCAGATCATGTTCGAGT	202	58
	RT-LOC113815142-R	GATGACTCAGATCATGTTCGAGT		
Primer-LOC113805465	RT-LOC113805465-F	CATCTTCCACTACCTACAACCTC	207	58
	RT-LOC113805465-R	TATGGGTGTGATGATGAATGGAG		
Primer-LOC113822686	RT-LOC113822686-F	CCACAGACAGTACATTAGTTCCA	204	58
	RT-LOC113822686-R	CCACAGACAGTACATTAGTTCCA		
Primer-LOC113812656	RT-LOC113812656-F	CTTACAACAAACTCTGCAGTGAC	249	58
	RT-LOC113812656-R	GTTACTCCATCATCTTCAGAGGG		
Primer-	RT-LOC113810364-F	CATTGGCAACCAGTTCAAGAC	225	57

LOC113810364	RT-LOC113810364-R	TTCTTGTCCTCATCAGTGCAG		
Primer-LOC113814611	RT-LOC113814611-F	GAAGATCATGAGGAACCTCTGG	220	59
	RT-LOC113814611-R	CTTGAACTGGTTGGCAATGAAA		
Primer-LOC113818538	RT-LOC113818538-F	TAACGTGGAGCCTATTTACACC	186	58
	RT-LOC113818538-R	TAAGATAGCCGGAGTAGGTACC		
Primer-LOC113825092	RT-LOC113825092-F	TGATGTCTCAGTGCTTCTGGT	192	58
	RT-LOC113825092-R	GATGTAGGTCCAGTTCCCCTC		
Primer-LOC113824295	RT-LOC113824295-F	TTAAATATGGCGAGTCTCGGGA	208	56
	RT-LOC113824295-R	CTGAAGCGTCTCATCGATCTTC		
Primer-LOC113807384	RT-LOC113807384-F	GCTGATTCTTCATCCGTCGAAT	171	57
	RT-LOC113807384-R	GGCGCCATATACGGATTGTAAT		
Primer-LOC113805646	RT-LOC113805646-F	CTCGACAACGCCAACTCCT	182	56
	RT-LOC113805646-R	TACAGGGCCGCGTAGTAGT		
Primer-	RT-LOC113815782-F	GCACTTTAACCCAACACTCAAG	176	56

LOC113815782	RT-LOC113815782-R	CACGTGAAGAAGGTACTGTTCT		
Primer-LOC113824427	RT-LOC113824427-F	GGAGAATTCCCATGGTTGGTAA	178	56
	RT-LOC113824427-R	CACCACCACTAGTTCTTGGATT		
Primer-LOC113822227	RT-LOC113822227-F	TGCATTTCAACAGGCTAGTCG	185	58
	RT-LOC113822227-R	GAAGTGGAGACCATCTGGACA		
Primer-LOC113822220	RT-LOC113822220-F	CCCCACTGTTGATAAGCAGC	194	56
	RT-LOC113822220-R	GCTGCACATGTAGAAGGAGC		

Table S4 List of pre-processing results of sequencing data

Sample	RawReads	RawBases	CleanReads	CleanBases	ValidBases	Q30	GC
Eh1_1	47.69M	7.15G	46.83M	6.86G	95.95%	93.44%	51.20%
Eh3_1	43.17M	6.48G	42.30M	6.21G	95.94%	93.58%	51.88%
Eh3_4	46.75M	7.01G	45.96M	6.76G	96.43%	93.68%	50.69%
Mh1_1	51.59M	7.74G	50.66M	7.44G	96.18%	93.59%	51.12%
Mh1_2	48.11M	7.22G	47.27M	6.95G	96.30%	93.65%	51.29%

Mh2_1	46.76M	7.01G	45.97M	6.75G	96.21%	93.80%	50.69%

Table S5 Statistical results of reads and reference genome alignment

Sample	Totalreads	Total mapped reads	Multiple mapped	Uniquely mapped	Read-1	Read-2	Reads map to '+'	Reads map to '-'	Non-splice reads	Splice reads	Reads mapped in proper pairs
Eh1_1	46833724	42307393(90.34%)	9730779(20.78%)	32576614(69.56%)	16314684(34.84%)	16261930(34.72%)	16309934(34.83%)	16266680(34.73%)	17031330(36.37%)	15545284(33.19%)	30498742(65.12%)
Eh3_1	42295220	38272190(90.49%)	9474680(22.40%)	28797510(68.09%)	14425256(34.11%)	14372254(33.98%)	14403604(34.05%)	14393906(34.03%)	14633698(34.60%)	14163812(33.49%)	27000674(63.84%)
Eh3_4	45962552	41683552(90.69%)	9132795(19.87%)	32550757(70.82%)	16297511(35.46%)	16253246(35.36%)	16282989(35.43%)	16267768(35.39%)	17166381(37.35%)	15384376(33.47%)	30532392(66.43%)
Mh1_1	50662346	45636213(90.08%)	9912072(19.56%)	35724141(70.51%)	17889923(35.31%)	17834218(35.20%)	17860487(35.25%)	17863654(35.26%)	18707566(36.93%)	17016575(33.59%)	33485532(66.10%)
Mh1_2	47274580	42596973(90.11%)	9391286(19.87%)	33205687(70.24%)	16623041(35.16%)	16582646(35.08%)	16616483(35.15%)	16589204(35.09%)	17339043(36.68%)	15866644(33.56%)	31037426(65.65%)
Mh2_1	45974640	41546657(90.37%)	9169456(19.94%)	32377201(70.42%)	16210156(35.26%)	16167045(35.17%)	16204691(35.25%)	16172510(35.18%)	17070419(37.13%)	15306782(33.29%)	30197870(65.68%)

Table S6 Top 30 GO terms of biological functions of up-regulated DEGs

id	Term	Category	ListHits	pValue
GO:0007443	Malpighian tubule morphogenesis	biological_process	3	1.96E-06

GO:0010165	response to X-ray	biological_process	3	3.49E-06
GO:0006030	chitin metabolic process	biological_process	5	6.75E-06
GO:0048699	generation of neurons	biological_process	3	1.90E-05
GO:0006936	muscle contraction	biological_process	5	2.91E-05
GO:0030239	myofibril assembly	biological_process	5	3.30E-05
GO:0003281	ventricular septum development	biological_process	3	7.72E-05
GO:0060976	coronary vasculature development	biological_process	3	7.72E-05
GO:0008585	female gonad development	biological_process	3	9.67E-05
GO:0035277	spiracle morphogenesis, open tracheal system	biological_process	3	9.67E-05
GO:0032982	myosin filament	cellular_component	4	0.000116292
GO:0009986	cell surface	cellular_component	10	0.000120917
GO:0016459	myosin complex	cellular_component	5	0.000208721
GO:0005581	collagen trimer	cellular_component	3	0.000296844
GO:0009925	basal plasma membrane	cellular_component	4	0.000299354
GO:0031672	A band	cellular_component	4	0.000367943

GO:0005886	plasma membrane	cellular_component	42	0.00044843
GO:0043235	receptor complex	cellular_component	5	0.000456475
GO:0005887	integral component of plasma membrane	cellular_component	21	0.000457824
GO:0005615	extracellular space	cellular_component	18	0.001221383
GO:0005201	extracellular matrix structural constituent	molecular_function	6	1.64E-05
GO:0005200	structural constituent of cytoskeleton	molecular_function	5	4.21E-05
GO:0005229	intracellular calcium activated chloride channel activity	molecular_function	4	4.83E-05
GO:0008307	structural constituent of muscle	molecular_function	4	0.000406388
GO:0030674	protein binding, bridging	molecular_function	3	0.000534898
GO:0005509	calcium ion binding	molecular_function	14	0.000709243
GO:0008201	heparin binding	molecular_function	4	0.001324042
GO:0030898	actin-dependent ATPase activity	molecular_function	3	0.001370317
GO:0008144	drug binding	molecular_function	3	0.001370317
GO:0005518	collagen binding	molecular_function	3	0.001515943

Table S7 Top 30 GO terms of biological functions of down-regulated DEGs

id	Term	Category	ListHits	pValue
GO:0006629	lipid metabolic process	biological_process	3	0.000127897
GO:0070062	extracellular exosome	cellular_component	5	0.000905345
GO:0005615	extracellular space	cellular_component	7	0.001004194
GO:0000139	Golgi membrane	cellular_component	5	0.002839137
GO:0016020	membrane	cellular_component	4	0.075853797
GO:0005576	extracellular region	cellular_component	6	0.133710757
GO:0005737	cytoplasm	cellular_component	10	0.305274966
GO:0005829	cytosol	cellular_component	5	0.57713756
GO:0016021	integral component of membrane	cellular_component	6	0.762356883
GO:0005886	plasma membrane	cellular_component	4	0.765513976
GO:0005634	nucleus	cellular_component	7	0.815072638
GO:0003677	DNA binding	molecular_function	3	0.242925058
GO:0005524	ATP binding	molecular_function	5	0.24347072
GO:0046872	metal ion binding	molecular_function	5	0.562390822

Table S8 KEGG analysis of up-regulated enrichment pathways

id	Term	ListHits	pValue	qValue
ko04530	Tight junction	11	5.24E-07	5.76E-05
ko04391	Hippo signaling pathway - fly	9	2.41E-06	0.0001326
ko04921	Oxytocin signaling pathway	9	3.98E-06	0.000146
ko04745	Phototransduction - fly	8	5.73E-06	0.0001575
ko04510	Focal adhesion	11	1.59E-05	0.000291
ko04145	Phagosome	10	1.34E-05	0.000291
ko04520	Adherens junction	8	2.81E-05	0.0004415
ko04670	Leukocyte transendothelial migration	7	3.32E-05	0.0004566
ko04611	Platelet activation	8	3.80E-05	0.0004641
ko04210	Apoptosis	9	4.66E-05	0.0004787
ko04919	Thyroid hormone signaling pathway	8	4.79E-05	0.0004787
ko04015	Rap1 signaling pathway	9	5.39E-05	0.0004944
ko04540	Gap junction	5	0.0001175	0.0009945
ko04610	Complement and coagulation cascades	3	0.0001378	0.0010825
ko00401	Novobiocin biosynthesis	1	0.0001543	0.0011317
ko04810	Regulation of actin cytoskeleton	8	0.0001897	0.0013042
ko00643	Styrene degradation	1	0.0004592	0.0029714
ko04390	Hippo signaling pathway	7	0.0005434	0.0033205
ko04974	Protein digestion and absorption	4	0.000908	0.0050106
ko00960	Tropane, piperidine and pyridine alkaloid biosynthesis	1	0.000911	0.0050106

ko00350	Tyrosine metabolism	2	0.0011223	0.0058787
ko00400	Phenylalanine, tyrosine and tryptophan biosynthesis	1	0.002241	0.0112048
ko04260	Cardiac muscle contraction	4	0.0024426	0.0116821
ko04261	Adrenergic signaling in cardiomyocytes	5	0.0032019	0.0140883
ko04918	Thyroid hormone synthesis	3	0.0031001	0.0140883
ko00950	Isoquinoline alkaloid biosynthesis	1	0.0041161	0.0174141
ko04912	GnRH signaling pathway	3	0.0060676	0.0247199
ko00130	Ubiquinone and other terpenoid-quinone biosynthesis	1	0.0078918	0.0310036
ko00360	Phenylalanine metabolism	1	0.0093943	0.0356334
ko00232	Caffeine metabolism	1	0.0127461	0.0467356
ko04512	ECM-receptor interaction	3	0.0133484	0.0473653
ko00534	Glycosaminoglycan biosynthesis - heparan sulfate / heparin	1	0.0165404	0.0568575
ko04924	Renin secretion	2	0.017203	0.0573433
ko00770	Pantothenate and CoA biosynthesis	1	0.0185961	0.0601639
ko04072	Phospholipase D signaling pathway	3	0.0237735	0.0747167
ko00240	Pyrimidine metabolism	3	0.0246642	0.0753627
ko04725	Cholinergic synapse	2	0.0267566	0.0795467
ko04060	Cytokine-cytokine receptor interaction	1	0.0278107	0.0805046
ko04310	Wnt signaling pathway	3	0.0294202	0.0829801
ko04146	Peroxisome	3	0.0346876	0.0953909
ko00760	Nicotinate and nicotinamide metabolism	1	0.0356876	0.0957471

ko04915	Estrogen signaling pathway	2	0.0402729	0.1054766
ko04213	Longevity regulating pathway - multiple species	3	0.0454754	0.1163324
ko00230	Purine metabolism	4	0.0478145	0.11688
ko04961	Endocrine and other factor-regulated calcium reabsorption	1	0.0473477	0.11688

Table S9 KEGG analysis of down-regulated enrichment pathways

id	Term	ListHits	pValue	qValue
ko00944	Flavone and flavonol biosynthesis	1	8.52E-05	0.00192647
ko00860	Porphyrin and chlorophyll metabolism	2	0.000178153	0.00192647
ko00511	Other glycan degradation	2	0.000192647	0.00192647
ko00061	Fatty acid biosynthesis	1	0.000380229	0.002809756
ko00600	Sphingolipid metabolism	2	0.000537122	0.002809756
ko00500	Starch and sucrose metabolism	2	0.000566491	0.002809756
ko00450	Selenocompound metabolism	1	0.00065561	0.002809756
ko00720	Carbon fixation pathways in prokaryotes	1	0.001003345	0.003762544
ko00670	One carbon pool by folate	1	0.001274793	0.003907174
ko04142	Lysosome	3	0.001302391	0.003907174
ko03060	Protein export	1	0.0022756	0.006206183
ko00531	Glycosaminoglycan degradation	1	0.00267024	0.006675599
ko04341	Hedgehog signaling pathway - fly	1	0.004812232	0.010899434
ko04920	Adipocytokine signaling pathway	1	0.005086403	0.010899434
ko03050	Proteasome	1	0.006878884	0.013291082

ko00071	Fatty acid degradation	1	0.007201842	0.013291082
ko01212	Fatty acid metabolism	1	0.007531613	0.013291082
ko00040	Pentose and glucuronate interconversions	1	0.008211443	0.013685738
ko03320	PPAR signaling pathway	1	0.009651179	0.015238704
ko00983	Drug metabolism - other enzymes	1	0.01041048	0.015428368
ko00260	Glycine, serine and threonine metabolism	1	0.010799857	0.015428368
ko04070	Phosphatidylinositol signaling system	1	0.015499597	0.021135814
ko04550	Signaling pathways regulating pluripotency of stem cells	1	0.021457845	0.027988493
ko00562	Inositol phosphate metabolism	1	0.022531114	0.028163893
ko00240	Pyrimidine metabolism	1	0.028823588	0.033954678
ko04020	Calcium signaling pathway	1	0.029427388	0.033954678

Table S10 The sequence information for phylogenetic tree of PAQR proteins

Sequence ID	annotation	species
NP_001292998.1	adiponectin receptor protein 1	*Mus musculus*
NP_001277486.1	adiponectin receptor protein 1	*Homo sapiens*
NP_001342621.1	adiponectin receptor protein 2	*Mus musculus*
NP_001362292.1	adiponectin receptor protein 2	*Homo sapiens*
NP_001346839.1	progestin and adipoQ receptor family member 3	*Mus musculus*
XP_019881240.1	progestin and adipoQ receptor family member 3	*Aethina tumida*

KDR18346.1	progestin and adipoQ receptor family member 3	*Zootermopsis nevadensis*
XP_051161254.1	progestin and adipoQ receptor family member 3	*Leptopilina boulardi*
XP_050665144.1	progestin and adipoQ receptor family member 3	*Leptidea sinapis*
XP_050070597.1	progestin and adipoQ receptor family member 3	*Anopheles maculipalpis*
XP_035434305.1	progestin and adipoQ receptor family member 3	*Spodoptera frugiperda*
KAI2534791.1	progestin and adipoQ receptor family member 3	*Homo sapiens*
XP_001628391.2	progestin and adipoQ receptor family member 3	*Nematostella vectensis*
XP_050692791.1	progestin and adipoQ receptor family member 3	*Eriocheir sinensis*
IOCAS.LVAN10696	progestin and adipoQ receptor family member 3	*Penaeus vannamei*
NP_001017377.1	progestin and adipoQ receptor family member 4	*Rattus norvegicus*
XP_050666962.1	progestin and adipoQ receptor family member 4	*Leptidea sinapis*
XP_050846650.1	progestin and adipoQ receptor family member 4	*Vespula vulgaris*
XP_050537260.1	progestin and adipoQ receptor family member 4	*Daktulosphaira vitifoliae*
KAI2574880.1	progestin and adipoQ receptor family member 5	*Homo sapiens*
KAF6393166.1	progestin and adipoQ receptor family member 5	*Pipistrellus kuhlii*

KAI4083195.1	progestin and adipoQ receptor family member 6	*Homo sapiens*
KAF6397522.1	progestin and adipoQ receptor family member 6	*Rousettus aegyptiacus*
KAI4079235.1	progestin and adipoQ receptor family member 7	*Homo sapiens*
KAF6383140.1	progestin and adipoQ receptor family member 7	*Pipistrellus kuhlii*
KAG6934155.1	progestin and adipoQ receptor family member 8	*Chelydra serpentina*
KAI4018636.1	progestin and adipoQ receptor family member 8	*Homo sapiens*
KAF6365023.1	progestin and adipoQ receptor family member 8	*Rhinolophus ferrumequinum*
KXJ22365.1	progestin and adipoQ receptor family member 9	*Exaiptasia diaphana*
KDR16600.1	progestin and adipoQ receptor family member 10	*Zootermopsis nevadensis*
GBM64126.1	progestin and adipoQ receptor family member 10	*Araneus ventricosus*
XM_027379195.1	Mmd2 X1	*Litopenaeus vannamei*
c83205_g1	Mmd2 X2	*Litopenaeus vannamei*
IOCAS.LVAN13454	adiponectin receptor protein 1	*Litopenaeus vannamei*
IOCAS.LVAN20755	progestin and adipoQ receptor family member 9-1	*Litopenaeus vannamei*
IOCAS.LVAN20754	progestin and adipoQ receptor family member 9-2	*Litopenaeus vannamei*
AAR08377.1	progestin and adipoQ receptor family member 11	*Homo sapiens*

AAR08388.1	progestin and adipoQ receptor family member 11	*Mus musculus*

2 Supplementary Figures

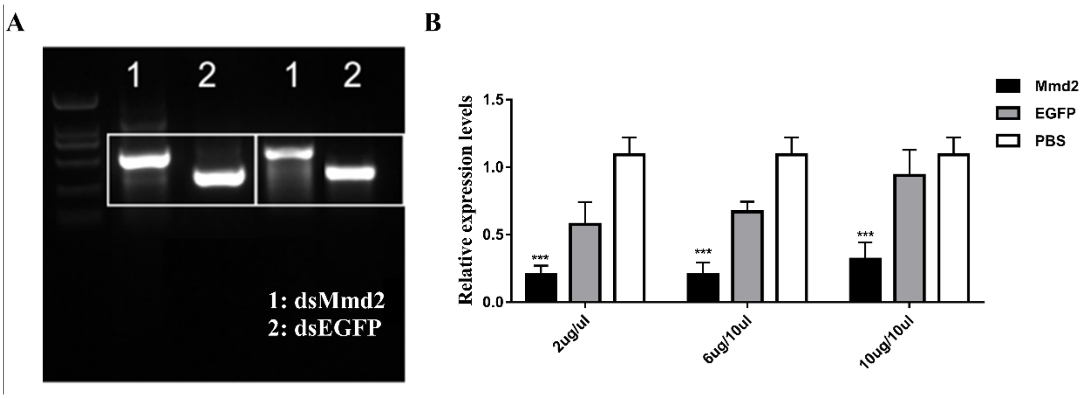

Supplementary Figure S1 Qualified dsRNA and the *LvMmd2* gene expression in optimization experiment with different dosages of double stranded RNA (dsRNA).

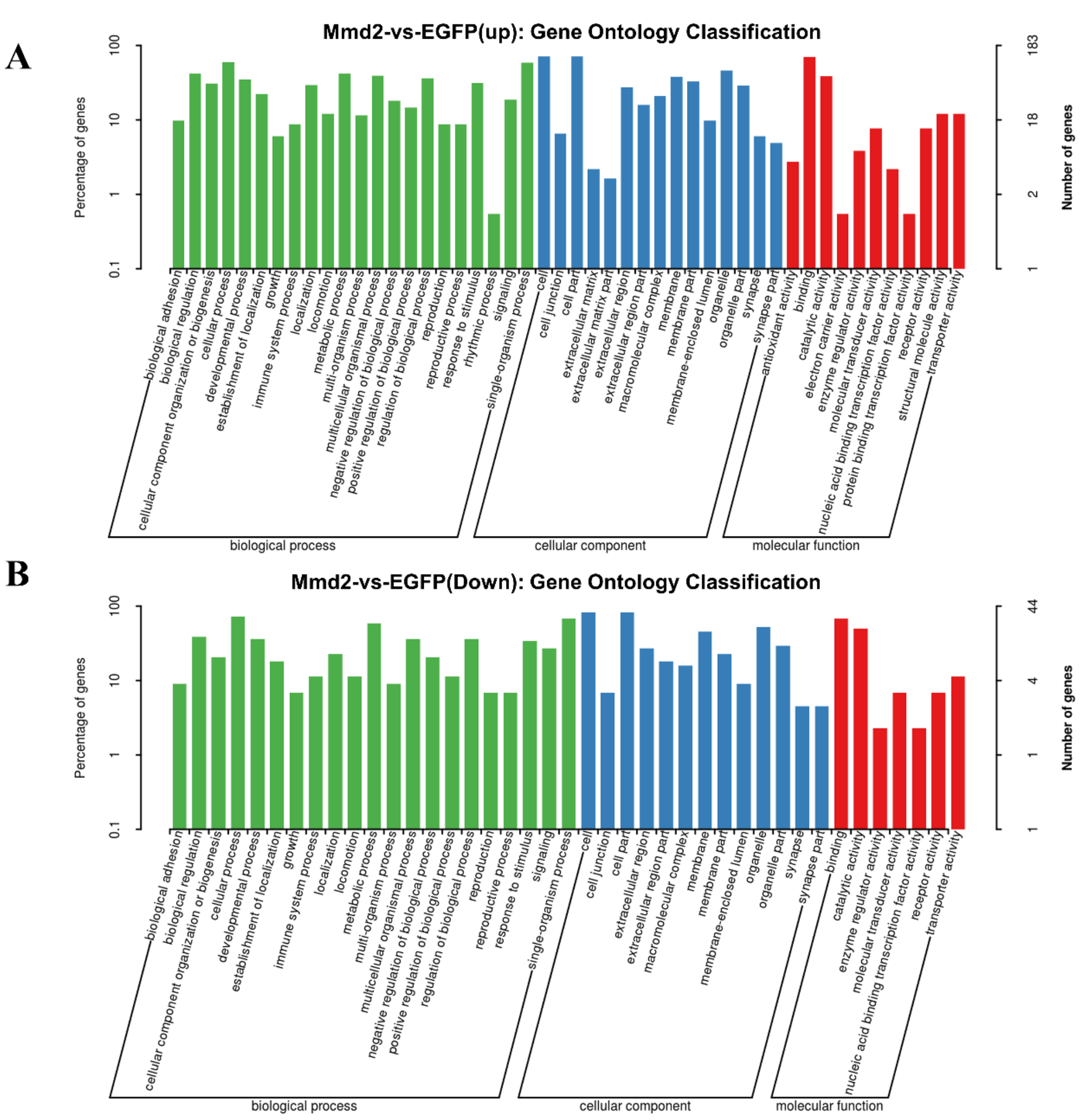

Supplementary Figure S2 Gene ontology functional classification of down-regulated and up-regulated DEGs. Gene ontology analysis of up-regulation gene. (B) Gene ontology analysis of down-regulation genes

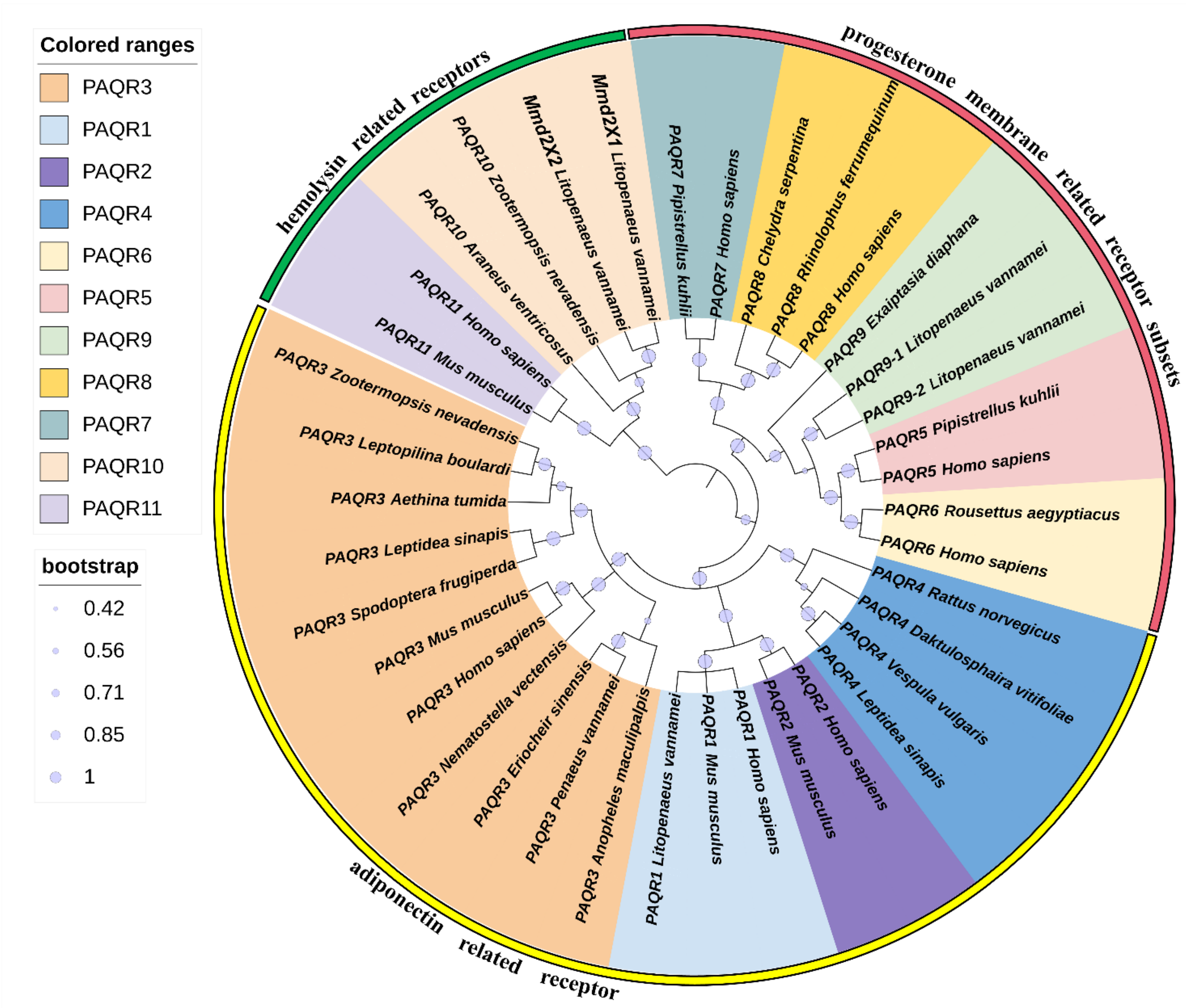

Supplementary Figure S3 Phylogenetic tree of PAQR proteins. The bootstrap values are given at each branch node. The red letters stand for *L. vannamei* PAQR10. The orange font represents the other PAQR family members of *L. vannamei* except PAQR10.
